# A Phase II Pilot Study of Anti‐PD‐L1, Durvalumab, and a PARP Inhibitor, Olaparib in Patients With Metastatic Triple‐Negative Breast Cancer With or Without Germline BRCA Mutation

**DOI:** 10.1002/cam4.71220

**Published:** 2025-12-08

**Authors:** Takeo Fujii, Ashley Cimino‐Mathews, Stanley Lipkowitz, Min‐Jung Lee, Jayakumar Nair, Britanny Brooke Solarz, Alexandra Zimmer, Bernadette Redd, Elliot B. Levy, Shraddha Rastogi, Nahoko Sato, Ann McCoy, Seth M. Steinberg, Jung‐Min Lee

**Affiliations:** ^1^ Women's Malignancies Branch, Center for Cancer Research, National Cancer Institute National Institutes of Health Bethesda Maryland USA; ^2^ Department of Pathology The Johns Hopkins University School of Medicine Baltimore Maryland USA; ^3^ Developmental Therapeutic Branch, Center for Cancer Research, National Cancer Institute National Institutes of Health Bethesda Maryland USA; ^4^ Radiology and Imaging Sciences, National Cancer Institute National Institutes of Health Bethesda Maryland USA; ^5^ Collaborative Biostatistics Section, Center for Cancer Research, National Cancer Institute National Institutes of Health Bethesda Maryland USA

**Keywords:** BRCA, clinical trials, durvalumab, olaparib, triple‐negative breast cancer

## Abstract

**Background:**

Immunostimulatory effects of PARP inhibitors could increase sensitivity to immune checkpoint inhibitors. The previous Phase II trial (MEDIOLA) reported clinical benefits of durvalumab and olaparib (D + O) in patients with germline *BRCA* ‐mutated (gBRCAm) HER2‐negative metastatic breast cancer. Yet, the clinical activity of D + O in germline *BRCA* wild‐type (gBRCAwt) triple‐negative breast cancer (TNBC) remains unknown.

**Methods:**

This single‐arm Phase II study tested D + O in patients with metastatic TNBC. The primary objective was overall response rate (ORR). Secondary objectives were safety, disease control rate (DCR), progression‐free survival (PFS) and overall survival (OS). Based on gBRCA status, patients were assigned to either gBRCAwt or gBRCAm cohort and were treated with D (1500 mg iv q4w) and O (300 mg twice a day orally). Pretreatment fresh tissues and serial blood samples were collected for correlative studies.

**Results:**

Fifteen patients (12 gBRCAwt and 3 gBRCAm) were enrolled. gBRCAm and gBRCAwt cohorts are reported as a combined dataset because of small sample size due to COVID‐19 and slow accrual. The median number of prior therapies was three (range 0–8). Among 14 RECIST‐evaluable patients (11 gBRCAwt and 3 gBRCAm), ORR was 28.6% (3 gBRCAm and 1 gBRCAwt). DCR was 64.3% (3 gBRCAm and 6 gBRCAwt). The median PFS and OS were 3.6 months (95% confidence interval [CI]: 1.8–5.7) and 10.7 months (95% CI: 5.9–38.9), respectively. There is one gBRCAm patient with ongoing durable PR (67.4+ months). There was no new safety concern. CD83 expression on Types 1 and 2 conventional dendritic cells in blood at baseline was low in the patients with PFS ≥ 4 months compared to those with PFS < 4 months.

**Conclusion:**

Our study demonstrated modest clinical benefits of D + O with ORR of 28.6% in subsets of heavily pretreated TNBC. Further detailed classification of DCs to understand the predictive role of DCs and prospective validation in a large cohort is required.

**Trial Registration:**

ClinicalTrials.gov identifier: NCT02484404

## 1 Introduction

Triple‐negative breast cancer (TNBC) accounts for approximately 15%–20% of all breast cancers and is defined as a lack of the expression of estrogen receptor (ER), progesterone receptor (PgR), and the amplification of the human epidermal growth factor 2 (HER2) receptor [1]. Among all the subtypes of breast cancer, metastatic TNBC has the worst survival outcomes with the median overall survival of 13–23 months [2, 3, 4]. Despite the recent advances in systemic therapies including immune checkpoint inhibitors (ICIs), the prognosis of TNBC remains poor and only a limited number of patients with metastatic TNBC derive clinical benefits from ICIs [3]. Hence, further investigations for novel therapeutic strategies are needed [3].

Currently, ICI in combination with chemotherapy is the standard of care therapy in advanced/metastatic TNBC [3]. Mechanistically, ICIs block the binding of checkpoint proteins (i.e., programmed cell death protein 1 [PD‐1] and cytotoxic T‐lymphocyte‐associated protein 4 [CTLA‐4]) to their ligands (i.e., programmed cell death protein ligand 1 [PD‐L1] and CD80/CD86, respectively), resulting in immune cell activation and attack of the cancer cells [5]. The KEYNOTE‐355 trial, one of the practice‐changing clinical trials for TNBC, demonstrated a significant overall survival (OS) benefit of adding the anti‐PD1 antibody, pembrolizumab, to chemotherapy compared with chemotherapy alone in patients with advanced TNBC whose tumors had a PD‐L1 combined positive score (CPS) ≥ 10 by immunohistochemistry (IHC) (median OS of 23 months in pembrolizumab + chemotherapy versus 16.1 months in chemotherapy alone). Based on this result, the US Food and Drug Administration (FDA) approved pembrolizumab in combination with chemotherapy for locally recurrent unresectable or metastatic TNBC with a positive PD‐L1 CPS defined as ≥ 10. However, it is noteworthy that only one third or less of TNBC tumors have a positive PD‐L1 CPS [3, 6], leaving two thirds of patients with metastatic TNBC ineligible for ICI and chemotherapy combination under the current indication.

Among novel therapies, poly ADP ribose polymerase (PARP) inhibitors (PARPi) have changed the treatment paradigm in TNBC with a germline *BRCA* 1/2 mutation (gBRCAm) and are approved by the FDA in both non‐metastatic and metastatic settings for gBRCAm tumors irrespective of breast cancer subtype [7, 8, 9]. In the EMBRACA trial, talazoparib significantly improved progression‐free survival (PFS) compared with chemotherapy alone in patients with gBRCAm and advanced breast cancer (median PFS of 8.6 months for talazoparib vs. 5.6 months for chemotherapy) [9]. Similarly, olaparib also demonstrated a PFS benefit compared to chemotherapy (median PFS of 7 months for olaparib vs. 4.2 months for chemotherapy) in patients with gBRCAm and metastatic HER2‐negative breast cancer [8]. The potential benefits of PARPi‐based therapy in other subgroups beyond those with gBRCAm need to be investigated.

The absence of effector lymphocytes in the tumor microenvironment (TME) represents a critical barrier to the efficacy of ICIs. To address this, substantial efforts have been directed toward developing therapeutic strategies aimed at increasing immune cell infiltration in TME [10]. Several preclinical studies have demonstrated immune‐stimulatory effects of PARPi in breast and ovarian cancer models. The activation of cGAS‐STING signaling by PARPi has been reported in a *BRCA* mutant TNBC cell line and in syngeneic and patient‐derived xenograft (PDX) mouse models [11, 12]. Similarly, in syngeneic immunocompetent mouse models of ovarian cancer and colon cancer lacking mutations in homologous recombination repair pathway genes, talazoparib, PARPi with potent PARP1 inhibitory activity comparable to olaparib, was reported to activate the cGAS‐STING pathway and augment the infiltration of CD8+ T cells into tumors, leading to the effective overcoming of the resistance to anti‐PD‐L1 antibody by combination treatment [13]. Additionally, PARPi also increases tumor mutational burden, resulting in enhanced sensitivity to ICIs in *BRCA* wild‐type TNBC cells [12]. In syngeneic mouse models of BRCA1‐deficient ovarian cancer and BRCA‐mutated TNBC cell lines, PARPi increases the infiltration of CD8+ T cells and NK cells in TME and upregulates PD‐L1 expression [14, 15, 16, 17]. These preclinical data suggest that the combination of PARPi and ICI may result in clinical activity in patients with gBRCAm TNBC as well as in those with germline *BRCA* wild‐type (gBRCAwt) TNBC.

Durvalumab is a human IgG1 kappa monoclonal antibody that inhibits PD‐L1's binding to the PD‐1 receptor to overcome the inhibition of primary human T‐cell activation [18]. The safety and activity of the combination of an ICI (durvalumab) and a PARPi (olaparib) were previously studied in a phase I trial [19] as well as in the single‐arm phase II trial (MEDIOLA trial) [20]. In the MEDIOLA trial, which enrolled patients with PARPi‐naive gBRCAm HER2‐negative metastatic breast cancer, a majority (87%) of the patients had one or two previous systemic therapies and only one‐third of the patients were treated with prior platinum agents [20]. In this study, we aimed to identify the clinical activity of the durvalumab and olaparib combination in heavily‐pretreated patients with metastatic TNBC, irrespective of germline *BRCA* mutation status (i.e., either gBRCAwt or gBRCAm). We also collected baseline fresh core biopsies and serial blood samples to investigate the immunomodulatory effects of the ICI + PARPi combination in patients with TNBC.

## 2 Methods

### 2.1 Study Design and Patient Selection

This single‐arm pilot Phase II trial of the combination of durvalumab and olaparib in patients with metastatic TNBC (ClinicalTrials.gov) was approved by the Institutional Review Board (IRB) of the Center for Cancer Research (CCR), National Cancer Institute (NCI). The study adhered to ethical principles derived from the Declaration of Helsinki and aligned with the International Council on Harmonization guidelines on Good Clinical Practice as well as all applicable laws, regulations, and all requirements by a regulatory authority and/or IRB. All patients provided written informed consent before enrollment in the study (Appendix S1).

Briefly, eligible patients had histologically confirmed metastatic or recurrent TNBC aged > 18 years. Patients were required to have a measurable disease determined by the Response Evaluation Criteria in Solid Tumors (RECIST) v1.1 and at least one lesion suitable for a mandatory baseline biopsy. The presence of germline *BRCA1* and *BRCA2* mutation status was evaluated prior to enrollment in the study. Additional major inclusion criteria included Eastern Cooperative Oncology Group (ECOG) performance status (PS) of 0–2 and adequate organ and marrow function. Prior treatment with ICIs except for durvalumab was allowed. Exclusion criteria included prior PARPi use, any investigational anticancer therapy within 3 weeks of the first doses of the study drugs, central nervous system metastases within 1 year before study enrollment, active or prior inflammatory bowel disease, and/or baseline signs of myelodysplastic syndrome or acute myelogenous leukemia. Details are shown in Data S1 in the Appendix S2. ER, PgR, and HER2 were assessed by the referring institution, and the pathology reports were used to determine eligibility criteria.

### 2.2 Procedures

Patients received durvalumab 1500 mg intravenously every 4 weeks and olaparib 300 mg twice daily orally (28‐day cycle) until the progression of disease, patient withdrawal, or intolerable toxicity. This recommended Phase 2 dose (RP2D) was determined in the Phase I trial, which we previously reported [19]. Dose delays and modifications were allowed according to the protocol (Data S2 in the Appendix S2).

Response was assessed using the RECIST v1.1. Imaging studies were performed every two cycles (approximately 8–9 weeks) unless clinically indicated in between imaging cycles. Adverse events (AEs) were assessed every 2 weeks during the first cycle and then every cycle by using Common Terminology Criteria for Adverse Events version 4.03 (CTCAE v.4.03).

For correlative studies, mandatory fresh core needle biopsies were performed before the administration of the study drug to assess tumoral PD‐L1 expression and tumor‐infiltrating lymphocytes. Serial (pretreatment and cycle 1 day 15 [C1D15] treatment) peripheral blood samples were collected for circulating tumor cells (CTCs) and systemic immune cell subset analysis.

### 2.3 Outcomes

The primary objective was to determine the overall response rate (ORR) by RECIST v1.1, defined as the rate of complete response (CR) and partial response (PR) at their best response. The secondary objectives included safety assessment using CTCAE v4.03, disease control rate (DCR) defined as the rate of CR, PR, and stable disease (SD) at their best response, progression‐free survival (PFS), and overall survival (OS). PFS was defined as the time from the date of Cycle 1 Day 1 treatment to the date of disease progression confirmed radiographically or clinically, or the date of death, whichever occurred first. OS was defined as the time from the date of Cycle 1 Day 1 treatment to the date of death from any cause.

### 2.4 PD‐L1 Expression and Stromal Tumor Infiltrating Lymphocytes (TILs)

#### 2.4.1 PD‐L1 IHC

The fresh core biopsy samples prior to treatment were formalin‐fixed and paraffin embedded. IHC was performed using the Leica Bond RX (Leica Biosystems). Slides were baked and dewaxed online, followed by antigen retrieval, blocking of endogenous peroxidase, and incubation with anti‐PD‐L1 antibody (clone SP142; abcam, cat#ab228462, lot#GR3224770‐29, concentration: 0.1 μg/mL) for 180 min at room temperature. The slides were then counterstained, dried, and coverslipped using Ecomount (Biocare Medical). Detection was achieved using a combination of the PowerVision (Leica) and Tyramide Signal Amplification (Akoya) systems.

PD‐L1 expression was evaluated on the tumor cells and immune cells located within the tumor‐associated stroma as previously described [19, 21]. The combined labeling of tumor cell and immune cell labeling was assessed using the CPS, defined as the total number of PD‐L1 positive immune cells (lymphocytes and macrophages) plus PD‐L1 positive tumor cells, divided by the total number of tumor cells, and multiplied by 100 [22, 23]. A CPS ≥ 10 was considered PD‐L1 positive.

#### 2.4.2 TILs Assessment

Hematoxylin and eosin (H&E) stained slides of biopsy samples prior to treatment were evaluated to confirm the presence of tumor and assess the level of TILs. Stromal TILs were quantified and scored as the percentage (0%–100%) of tumor stroma area occupied by mononuclear inflammatory cells, as previously described [19]. Areas with necrosis, acute inflammation, and non‐tumor stroma were excluded from the assessment. TILs ≥ 1 were considered TIL positive.

### 2.5 CTCs Analysis

Ten milliliter of peripheral blood samples were collected in an EDTA tube from each patient. Following red blood cell lysis, the remaining blood cells were incubated with a nuclear dye, Hoechst 33342 (Life Technologies), a viability dye, LIVE/DEAD Fixable Aqua (Life Technologies) and antibodies including PE‐conjugated anti‐human epithelial cell adhesion molecule (EpCAM) antibody (Miltenyi Biotec; clone HEA‐125). The anti‐PE magnetic beads (Miltenyi Biotec) were then used to isolate EpCAM‐positive cells [24, 25]. Viable, nucleated, EpCAM‐positive, CD45 (BioLegend; clone HI30)‐negative cells were identified and enumerated as CTCs using multiparameter flow cytometry (MACSQuant; Miltenyi Biotec) [26]. These CTCs were further characterized for the expression of CD117 (BioLegend; clone 104D2), C‐X‐C motif chemokine receptor 4 (CXCR4) (BioLegend; clone 12G5), PD‐L1 (BioLegend; clone 29E.2A3), and mucin‐1 (MUC‐1) (BD Biosciences; clone HMPV). Five distinct types of CTCs were identified as previously described [26, 27, 28, 29, 30]: conventional epithelial CTCs (EpCAM+ CTCs), stem cell‐like CTCs (CD117+ CTCs), CXCR4+ CTCs, PD‐L1+ CTCs, and MUC1+ CTCs.

### 2.6 Immune Cell Profiling by Flow Cytometry

PBMCs were isolated and viably frozen for storage until analysis. After thawing, they were washed with PBS, incubated with Fc receptor blocking reagent (Miltenyi Biotec), and stained with monoclonal antibodies for 20–30 min at 4°C. The viability dye, LIVE/DEAD Fixable Aqua (Life Technologies) was used to exclude dead cells. Multiparametric flow cytometry (MACSQuant; Miltenyi Biotec) was used for all assays. CD14 and CD16 were used to identify three different subsets of monocytes. CD3 was used to gate T cells, and then T cells were further categorized as CD4+ T cells and CD8+ T cells. A list of the other immunophenotypic markers and antibodies used is provided in Tables S1 and S2. FlowJo software v.10.6.1 (FlowJo LLC) was used for all the data analysis.

### 2.7 Statistical Methods

Considering the patients with gBRCAm may have a higher response rate (RR) to the combination treatment due to underlying homologous recombination deficiency [31, 32] and more immunogenic TME [33, 34] compared to the patients with gBRCAwt, those with gBRCAm and gBRCAwt were enrolled separately. For the gBRCAwt cohort, 16 evaluable patients were to be enrolled in the first stage based on Simon's minimax two‐stage design to rule out 10% RR and target 30% RR with 0.10 one‐sided alpha and 0.9 power. If there are two or more RECIST responses in the first stage, 9 additional patients were to be enrolled for the second stage. Five or more RECIST responses in 25 patients would warrant further studies. For the gBRCAm cohort, a small single‐stage design with a stopping rule was used because of the limited number of patients who might have been enrolled, given that gBRCAm TNBC accounts for 10%–20% of TNBC [35]. If one or more patients had a RECIST response in the first five patients, an additional five patients were to be enrolled.

The safety profile was assessed in all the patients who received at least one dose of study drugs. Kaplan–Meier curves for PFS and OS were generated, and median PFS and OS with 95% confidence intervals (95% CI) were calculated. Patients who were still alive at the date of the last follow‐up were censored. The log‐rank test was used to assess differences in PFS and OS between the gBRCAm and gBRCAwt groups. The group with durable clinical benefit (benefit group; *n* = 6) was defined as a group of patients with PFS ≥ 4 months. The remaining patients without durable clinical benefit were separately grouped (no benefit group; *n* = 9).

For correlative studies, we used the Wilcoxon matched‐pairs signed‐rank test to compare baseline to C1D15 values and the Mann–Whitney *U*‐test to compare the benefit group to the no benefit group at baseline. The associations between PD‐L1 or TILs and survival outcomes were assessed by using the univariate Cox proportional hazards model.

## 3 Results

### 3.1 Patient Characteristics

Between July 2015 and March 2020, a total of 15 patients (12 gBRCAwt and 3 gBRCAm) were enrolled and received at least one dose of each study drug. The data cutoff date was May 20, 2024. The enrollment was prematurely closed due to COVID‐19 and slow accrual. The participants from the gBRCAm and gBRCAwt cohorts are therefore reported together as a combined dataset along with the correlative study endpoints to have a reasonable sample size for analysis.

Median age was 50 (range 30–77). A cohort of patients in this study was heavily pretreated with a median number of three prior therapies (range 0–8), and eight (53.3%) patients had more than three lines of systemic treatments. Nine (60%) patients had prior platinum‐based therapy (Table 1). Of note, four (26.7%) patients had either hormone receptor+/HER2‐ breast cancer (*n* = 3) or hormone receptor+/HER2+ breast cancer (*n* = 1) at the initial diagnosis, which then changed to TNBC at recurrence or during the course of treatment for metastatic disease. The three patients with gBRCAm had deleterious mutations in either *BRCA1* (*n* = 2) or *BRCA*2 (*n* = 1). One patient with germline *BRCA2* mutation had a concurrent germline *NF1* mutation. Another patient with gBRCAwt had germline *MUTYH* mutation. The detailed germline DDR gene mutation status is summarized in Table 2.

**TABLE 1:** Baseline patient characteristics.

Characteristics	Patients (*N* = 15) (%)
Age, years, median (range)	50 (30–77)
Race	
White	9 (60)
Black	6 (40)
BRCA deleterious germline mutation status	
BRCA 1	2 (13.3)
BRCA 2	1 (6.7)
No mutation	12 (80)
ECOG performance status	
0	3 (20)
1	12 (80)
Prior platinum therapy	
Yes	9 (60)
No	6 (40)
Lines of prior systemic therapy	
0–2	7 (46.7)
≥ 3	8 (53.3)

Abbreviation: ECOG, Eastern Cooperative Oncology Group.

**TABLE 2:** Patient demographics, treatment response, and DNA damage repair gene mutations.

Study ID number	Demographics						Treatment response		DDR gene mutations	
	Age	Race	ECOG PS	Prior platinum	Lines of prior systemic therapies	Histology	RECIST response, clinical scenario	PFS (months)	Pathogenic germline mutations	Test type [# genes analyzed] (year of testing)
1012022	53	AA	1	No	4	IDC	PD, PD after 4 cycles without response	3.7	No	Ambry Genetics [BRCA1 and BRCA2 only] (2015)
1012062	30	AA	1	No	1	IDC	PR, new lesions after 23 cycles (maximum response −100% from baseline)	22.4	BRCA2 c.2092delC (p.Leu698TyrfsX32), NF1 c.5242C>T (p.Arg1748Ter)	GeneDx Custom Panel [9 genes] (2016)
1012069	48	W	1	Yes	2	IDC	PD, clinical PD with worsening symptoms and enlarging axillary lymph nodes during cycle 1	0.9	No	GeneDx Comprehensive Cancer Panel [32 genes] (2017)
1012081	54	W	1	Yes	2	IDC	PD, PD after 2 cycles without response	2.0	No	MYRIAD Integrated BRCAnalysis [25 genes] (2016)
1012086	33	AA	1	Yes	3	IDC	PD, PD after 2 cycles without response	2.0	No	INVITAE [42 genes] (2016)
1012092	50	AA	1	Yes	2	SCC	PD, PD after 2 cycles without response	1.5	No	MYRIAD Integrated BRCAnalysis [BRCA1 and BRCA2 only] (2016)
1012098	44	W	0	No	2	Poor. dif. IDC	PD, PD after 2 cycles without response	1.8	No	MYRIAD Integrated BRCAnalysis [BRCA1 and BRCA2 only] (2015)
1012100	77	AA	1	No	3	IDC	SD, initial response with SD for 9 cycles (maximum response −8.3% from baseline) and PD after 10 cycles	10.5	No	INVITAE [42 genes] (2017)
1012102	35	W	0	No	0	Metaplastic	PR, initial response with PR for 10 cycles (maximum response −52.2% from baseline) and then PD after 12 cycles	10.3	No	MYRIAD Integrated BRCAnalysis [Unknown] (2015)
1012112	60	AA	0	Yes	6	Poor. dif. IDC	PD, PD after 4 cycles with limited response after 2 cycles	3.8	No	MYRIAD Integrated BRCAnalysis [25 genes] (2015)
1012120	57	W	1	Yes	6	IDC	SD, initial response with SD for 4 cycles (maximum response −20% from baseline) and PD after 6 cycles with new lesions	5.8	No	GeneDx Breast/Ovarian Cancer Panel [21 genes] (2016)
1012126	72	W	1	No	3	Poor. dif. IDC	PD, clinical PD with worsening chest wall lesion and cancer‐related pain during cycle 3	3.2	No	GeneDx Breast Cancer Management Panel [9 genes] (2018)
1012139	56	W	1	Yes	8	IDC	PD, PD after 2 cycles without response	1.9	MUTYH c.933 + 3A>C, MUTYH c.1187G>A (p.Gly396Asp)	MYRIAD Integrated BRCAnalysis [25 genes] (2014)
1012153^a^	37	W	0	Yes	1	IDC	PR, exceptional responder with PR and remains on the treatment after 61 cycles as of May, 2024 (maximum response −99.9% from baseline)	67.4+	BRCA1 c.815_824dup10 (p.Thr276AlafsX14)	GeneDx Comprehensive Cancer Panel [32 genes] (2016)
1012159	37	W	1	Yes	4	IDC	PR, initial response with PD for 4 cycles (maximum response −66.7% from baseline) and PD after 6 cycles	4.8	BRCA1 c.68_69delAG	Ambry Genetics [BRCA1 and BRCA2 only] (2016)

*Note:* GeneDx Custom Panel [9 genes]: ATM, BRCA1, BRCA2, CDH1, CHECK2, NF1, PALB2, PTEN, TP53. GeneDx Comprehensive Cancer Panel [32 genes]: APC, ATM, AXIN2, BARD1, BMPR1A, BRCA1, BRCA2, BRIP1, CDH1, CDK4, CDKN2A, CHEK2, EPCAM, FANCC, MLH1, MSH2, MSH6, MUTYH, NBN, PALB2, PMS2, POLD1, POLE, PTEN, RAD51C, RAD51D, SCG5/GREM1, SMAD4, STK11, TP53, VHL, XRCC2. MYRIAD Integrated BRCAnalysis [25 genes]: APC, ATM, BARD1, BMPR1A, BRCA1, BRCA2, BRIP1, CDH1, CDK4, CDKN2A, CHECK2, EPCAM (large rearrangement only), MLH1, MSH2, MSH5, MUTYH, NBN, PALB2, PMS2, PTEN, RAD51C, RAD51D, SMAD4, STK11, TP53. INVITAE [42 genes]: APC, ATM, AXIN2, BARD, BMPR1A, BRCA1, BRCA2, BRIP1, CDH1, CDKN2A, CHECK2, DICER1, EPCAM (Deletion/duplication testing only), GREM1 (promoter region deletion/duplication testing only), KIT, MEN1, MLH1, MSH2, MSH6, MUTHY, NBN, NF1, PALB2, PDGFRA, PMS2, POLD1, POLE, PTEN, RAD50, RAD51C, RAD51D, SDHB, SDHC, SDHD, SMAD4, SMARCA4, STK11, TP53, TSC1, TSC2, VHL, SDHA (sequence change only). GeneDx Breast/Ovarian Cancer Panel [21 genes]: ATM, BARD1, BRCA1, BRCA2, BRIP1, CDH1, CHEK2, EPCAM, FANCC, MLH1, MSH2, MSH6, NBN, PALB2, PMS2, PTEN, RAD51C, RAD51D, STK11, TP53, XRCC2. GeneDx Breast Cancer Management Panel [9 genes]: ATM, BRCA1, BRCA2, CSH1, CHEK2, NBN, PALB2, PTEN, TP53.

Abbreviations: AA, African American; DDR, DNA damage repair; ECOG, Eastern Cooperative Oncology Group; IDC, invasive ductal carcinoma; poor. dif., poorly differentiated; PD, progressive disease; PFS, progression‐free survival; PR, partial response; PS, performance status; RECIST, Response Evaluation Criteria in Solid Tumors; SCC, squamous cell carcinoma; SD, stable disease; W, White.

^a^ Remain on treatment as of cutoff date of May 20, 2024.

### 3.2 Efficacy

**FIGURE 1:**
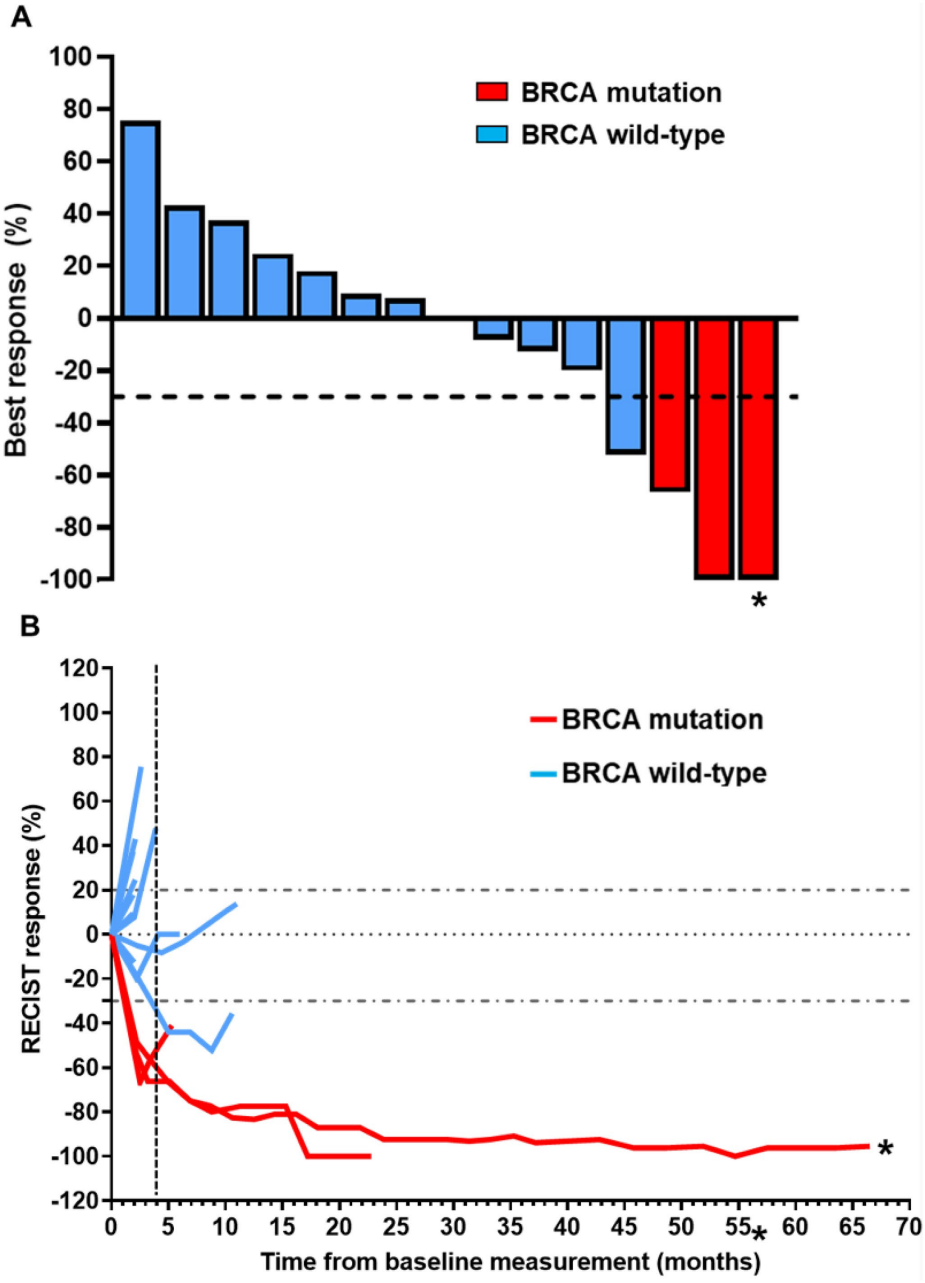
Best response to durvalumab and olaparib. (A) waterfall plot; (B) spider plot. *Exceptional responder.

**FIGURE 2:**
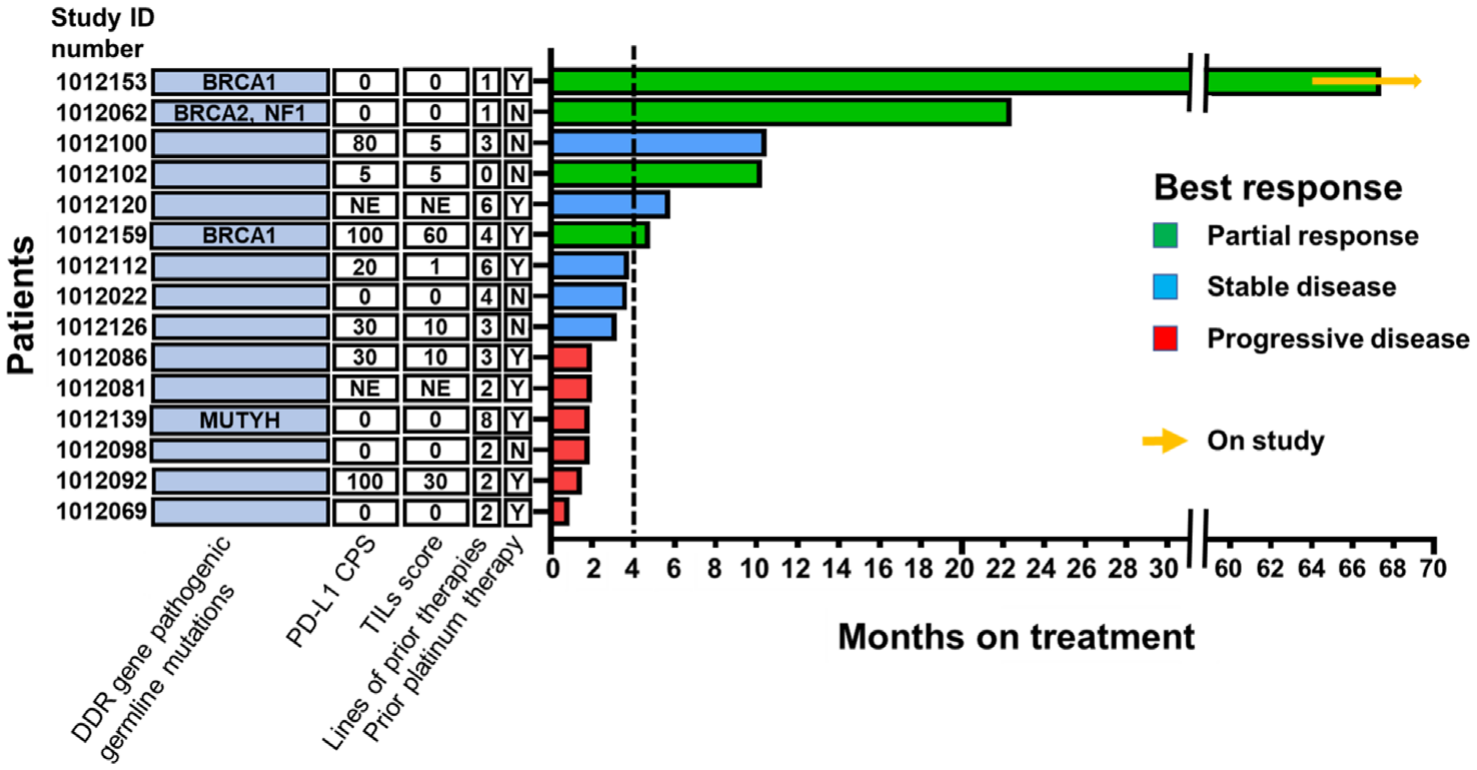
Swimmer plot. CPS, combined positive score; DOR, DNA damage repair; MUTYH, mutY DNA glycosylase; N, No; NE, not evaluable; NF1, Neurofibromatosis type 1; PD‐L1, Proqrammed death liqand 1; Tlls, tumor infiltratinq lymphocytes; Y, Yes.

**FIGURE 3:**
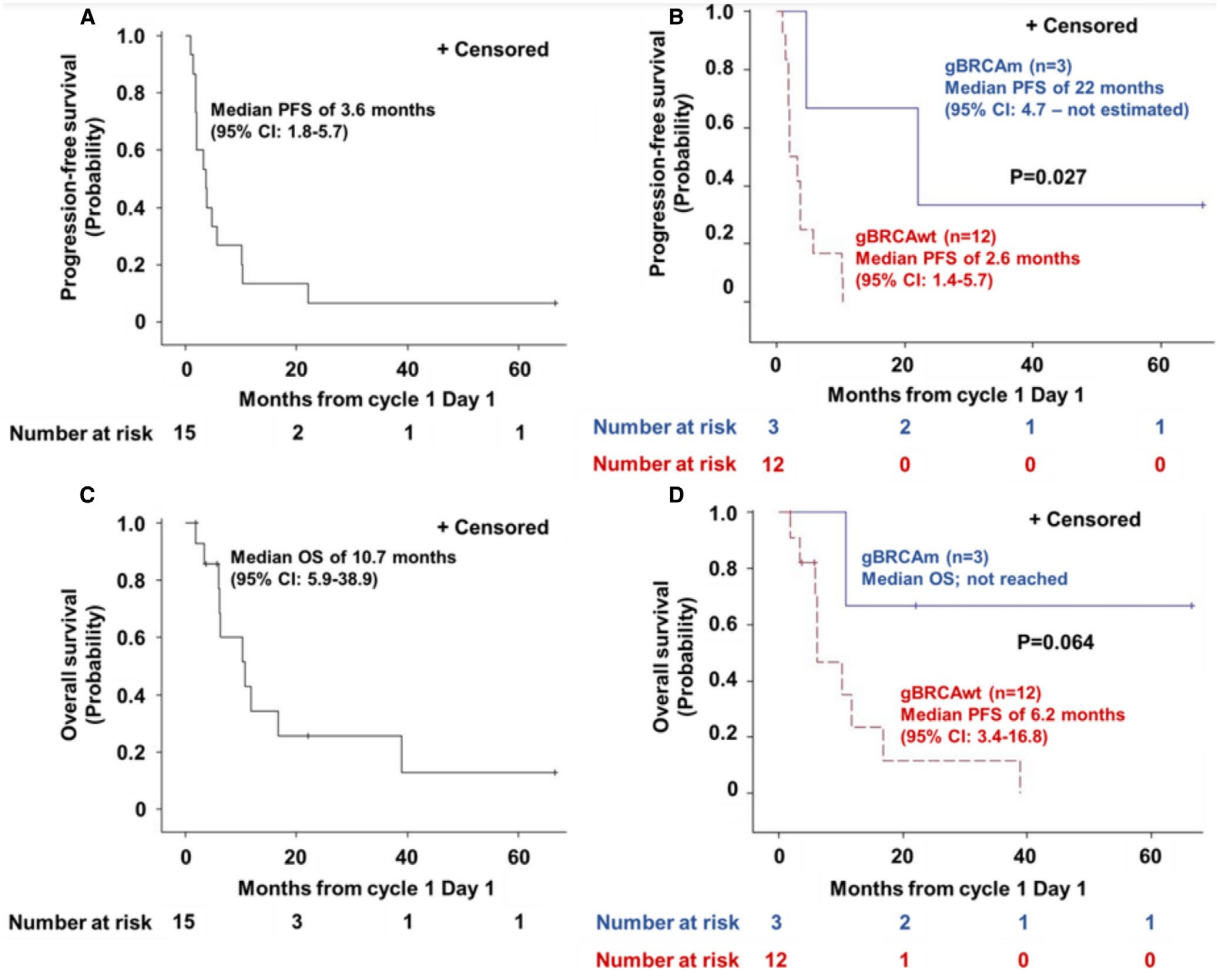
Kaplan–Meier estimates. (A) Progression‐free survival for the entire cohort. (B) Progression‐free survival for gBRCAm and gBRCAwt groups. (C) Overall survival for the entire cohort. (D) Overall survival for gBRCAm and gBRCAwt groups.

Of the 14 RECIST‐evaluable patients, ORR was 28.6% (4 of 14 patients; 3 gBRCAm and 1 gBRCAwt). DCR was 64.3% (9 of 14 patients; 3 gBRCAm and 6 gBRCAwt) (Figure 1A). One patient with gBRCAwt developed rapid clinical progression with worsening fatigue, elevated liver enzymes, and enlarging axillary lymph nodes during Cycle 1, thus not evaluable for RECIST response. All three patients with gBRCAm had PR (duration on the study as 4.8 months, 22.4 months, and 67.4+ months at the time of data cutoff) (Figures 1B, 2). In contrast, only one gBRCAwt patient achieved PR (10.3 months) (Figure 2) who initially presented with early stage metaplastic TNBC, underwent lumpectomy and adjuvant radiation without chemotherapy per patient's choice, then developed metastatic recurrence prior to the study enrollment. Of all 15 intention‐to‐treat patients, the median PFS and OS were 3.6 months (95% CI: 1.8–5.7) and 10.7 months (95% CI: 5.9–38.9), respectively (Figure 3A,C). PFS probability at 6 months was 26.7% (95% CI: 8.3–49.6) and OS probability at 12 months was 34.3% (95% CI: 10.7–59.9). Among 12 patients with gBRCAwt, the median PFS was 2.6 months (95% CI:1.4–5.7), significantly shorter than that observed among 3 patients with gBRCAm (median PFS 22 months [95% CI: 4.7‐not estimated], *p* = 0.027). The PFS probability at 6 months was 16.7% (95% CI: 2.7–41.3) in the gBRCAwt group compared to 66.7% in the gBRCAm group (95% CI: 2.7–41.3) (Figure 3B). The median OS in the gBRCAwt group was 6.2 months (95% CI: 3.4–16.8), numerically shorter than that in the gBRCAm group (median OS not reached, *p* = 0.064). The OS probability at 12 months was 23.4% (95% CI: 3.6–53.0) in the gBRCAwt group compared to 66.7% in the gBRCAm group (95% CI: 5.4–94.5) (Figure 3D).

### 3.3 Safety

No new safety signals were found. Most common AEs were mild and well tolerated (Table 3). There was no treatment discontinuation due to AE. Nine patients died from progressive disease during the follow‐up in the study, which was a minimum of 90 days after the final infusion of durvalumab, or 30 days after the final dose of olaparib, whichever occurs later, or until the initiation of an alternative cancer therapy according to the trial protocol.

**TABLE 3:** Treatment‐related adverse events by maximum grade per patient.

Adverse events	Any grade (%)	Grade 1 (%)	Grade 2 (%)	Grade 3 (%)	Grade 4 (%)
Hematologic					
Anemia	10 (66.7)	2 (13.33)	2 (13.33)	6 (40)	0
Decreased platelets	6 (40)	4 (26.7)	1 (6.67)	1 (6.67)	0
Decreased leukocytes	9 (60)	5 (33.3)	2 (13.33)	2 (13.33)	0
Decreased lymphocytes	11 (73.3)	2 (13.33)	4 (26.7)	4 (26.7)	1 (6.67)
Decreased neutrophils	4 (26.7)	1 (6.67)	1 (6.67)	2 (13.33)	0
Gastrointestinal					
Nausea	11 (73.3)	8 (53.3)	3 (20)	0	0
Vomiting	6 (40)	4 (26.7)	2 (13.33)	0	0
Diarrhea	3 (20)	3 (20)	0	0	0
Anorexia	4 (26.7)	4 (26.7)	0	0	0
GERD	3 (20)	2 (13.33)	1 (6.67)	0	0
Abdominal pain	1 (6.67)	1 (6.67)	0	0	0
Endocrinology and chemistry					
Hypothyroidism	1 (6.67)	0	1 (6.67)	0	0
Increased creatinine	6 (40)	6 (40)	0	0	0
Increased ALT/AST	4 (26.7)	2 (13.33)	2 (13.33)	0	0
Increased ALP	1 (6.67)	1 (6.67)	0	0	0
Hypocalcemia	3 (20)	3 (20)	0	0	0
Hypercalcemia	1 (6.67)	1 (6.67)	0	0	0
Hyponatremia	1 (6.67)	1 (6.67)	0	0	0
Hypokalemia	2 (13.33)	2 (13.33)	0	0	0
Hypomagnesemia	1 (6.67)	1 (6.67)	0	0	0
Hypophosphatemia	2 (13.33)	0	1 (6.67)	1 (6.67)	0
Hypoalbuminemia	1 (6.67)	0	1 (6.67)	0	0
Dermatologic					
Dry skin	1 (6.67)	1 (6.67)	0	0	0
Rash	2 (13.33)	1 (6.67)	0	1 (6.67)	0
Infusion related reaction	1 (6.67)	1 (6.67)	0	0	0
Others					
Fatigue	7 (46.7)	5 (33.3)	2 (13.33)	0	0
Dehydration	3 (20)	1 (6.67)	2 (13.33)	0	0
Dyspnea	3 (20)	2 (13.33)	0	1 (6.67)	0
Headache	3 (20)	3	0	0	0
Cough	3 (20)	2 (13.33)	0	1 (6.67)	0
Dizziness	2 (13.33)	2 (13.33)	0	0	0
Extremity edema	2 (13.33)	2 (13.33)	0	0	0
Oral mucositis	2 (13.33)	1 (6.67)	1 (6.67)	0	0
Fever/Chills	2 (13.33)	2 (13.33)	0	0	0
Depression	1 (6.67)	0	1 (6.67)	0	0
Dysgeusia	1 (6.67)	1 (6.67)	0	0	0
Dyspepsia	1 (6.67)	1 (6.67)	0	0	0
Dysphagia	1 (6.67)	1 (6.67)	0	0	0
Lung infection	1 (6.67)	0	1 (6.67)	0	0
Oral pain	1 (6.67)	1 (6.67)	0	0	0
Paresthesia	1 (6.67)	1 (6.67)	0	0	0
Weight loss	1 (6.67)	1 (6.67)	0	0	0
Wheezing	1 (6.67)	1 (6.67)	0	0	0

Abbreviations: ALP, alkaline phosphatase; ALT, alanine transaminase; AST, aspartate aminotransferase; GERD, gastroesophageal reflux disease.

### 3.4 Pretreatment PD‐L1 Expression and TILs

Thirteen of 15 patients (12 RECIST‐evaluable patients) had pretreatment fresh tumor biopsy samples with adequate quality and sufficient tumor cellularity for histologic and immunohistochemical analysis. Six (46.2%) patients had PD‐L1‐positive tumors; two of them had either durable SD for 10.5 months or PR for 4.8 months (Figure 2). The median PD‐L1 CPS in the benefit group (*n* = 5) and the no benefit group (*n* = 8) was 5 (range 0–100) and 10 (range 0–100), respectively (*p* = 0.74). Neither PD‐L1 CPS as a continuous variable nor PD‐L1 status as a binary variable was associated with PFS (PD‐L1 CPS, hazard ratio [HR] 1.003, 95% CI: 0.99–1.02, *p* = 0.7; PD‐L1‐positive vs. negative, HR 1.52, 95% CI: 0.46–5.1, *p* = 0.5) or OS (PD‐L1 CPS, HR 1.002, 95% CI: 0.99–1.02, *p* = 0.83; PD‐L1‐positive vs. negative, HR 1.95, 95% CI: 0.47–8.16, *p* = 0.36).

Seven (53.8%) tissue evaluable patients' tumors contained TILs (Figure 2). The median TILs for the benefit group (*n* = 5) and no benefit group (*n* = 8) were 5 (range 0–60) and 0.5 (range 0–30), respectively (*p* = 0.81). Neither TILs as a continuous variable nor binary variable was associated with PFS (TILs score, HR 1.001 95% CI: 0.98–1.04, *p* = 0.68; TILs present vs. absent, HR 1.35 95% CI: 0.39–4.72, *p* = 0.64) or OS (TILs score, HR 1.01 95% CI: 0.97–1.05, *p* = 0.59; TILs present vs. absent, HR 1.96 95% CI: 0.4–9.72, *p* = 0.41).

### 3.5 CTCs

There was no statistical association of the numbers of EpCAM+ CTCs or four subtypes of EpCAM+ CTCs at baseline with the benefit (PFS ≥ 4 months) or no benefit (PFS < 4 months) (Figure S1). Also, CTC counts did not change greatly on C1D15 compared to the baseline in either the benefit or no benefit group.

### 3.6 Peripheral Immune Subsets

The expression of the dendritic cell (DC) maturation marker, CD83, on both type 1 conventional DCs (cDC1s) [CD141+ DCs] and Type 2 conventional DCs (cDC2s) [CD1c + DCs] at baseline in the benefit group (PFS ≥ 4 months) was low compared to the no benefit group (PFS < 4 months) (*p* = 0.046 and 0.03, respectively) (Figure S2A,C). No substantial changes between baseline and C1D15 were observed (Figure S2B,C). Notably, the frequency of CD303+ plasmacytoid DCs increased (*p* = 0.031) at C1D15 only in the benefit group (Figure S3B). Additionally, the frequency of non‐classical monocytes among total viable cells was greatly decreased (*p* = 0.004) only in the no benefit group (Figure S3A). Also, in the no benefit group, the percentage of Ki67 negative (Ki67‐) inducible T cell co‐stimulator (ICOS) + CD8+ T cells increased (*p* = 0.027) while HLA‐DR+ Ki67‐positive (Ki67+) CD4+ T cells as well as effector CD4+ T cells decreased at C1D15 (Figure S4B–D, respectively). The benefit group showed the increase of the proportion of Ki67‐ PD‐L1+ CD8+ T cells in all CD8+ T cells on treatment compared to baseline (*p* = 0.031) (Figure S4A).

## 4 Discussion

Preclinical studies have supported the potential use of PARPi as an immunostimulatory agent in combination with ICIs through the activation of cGAS‐STING signaling [11, 12] and the increase of tumor mutational burden in TNBC with either gBRCAm or gBRCAwt [12]. Based on these findings, we hypothesized that the combination of PARPi and ICI would result in clinical benefit in patients with gBRCAwt metastatic TNBC. In our pilot phase II study, the combination of anti‐PD‐L1, durvalumab, and PARPi, olaparib, exhibited modest clinical activity with ORR of 28.6% (specifically, 8.3% ORR in gBRCAwt) in patients with heavily pretreated metastatic TNBC. The median PFS was significantly longer in the gBRCAm group than in the gBRCAwt group (22 months and 2.6 months, respectively, *p* = 0.027).

Although the same combination of durvalumab and olaparib was previously tested in the MEDIOLA trial and reported a higher ORR (59%, 10 out of 17 gBRCAm TNBC patients) [20], it is noteworthy that the present study enrolled more heavily pretreated patients (13% in MEDIOLA vs. 53.3% in the current study for those who had ≥ 3 prior systemic treatment regimens) and treated mixed gBRCAm and gBRCAwt populations which may partly explain this limited activity. The TOPACIO trial tested the combination of niraparib and pembrolizumab in 55 patients with metastatic TNBC, including both gBRCAm and gBRCAwt [36]. In 47 RECIST‐evaluable patients, the ORR was 21% (10 of 47 patients). Among the 20 patients with mutations in *BRCA* or other Homologous Recombination Repair (HRR) pathway genes, both somatic and germline, the ORR was notably higher at 40% (8 of 20 patients). Conversely, the ORR for the 22 patients with HRR wild‐type was only 9.1% (2 of 22 patients). These findings align with our observations regarding the ORR in the mixed population of gBRCAm and gBRCAwt patients and the enhanced clinical benefit observed in the gBRCAm group.

Also, we report the exceptional responder who currently remains on the study with PR of more than 5 years. This is a clinically meaningful outcome even among gBRCAm patients, given that olaparib monotherapy demonstrated a median PFS of 7 months in TNBC patients in the OlympiAD trial [8] suggesting the potential long‐term benefit of adding ICI to PARPi. It is, however, unclear as to why she attained a durable clinical response to the combination therapy. This exceptional responder received neoadjuvant platinum‐based chemotherapy followed by mastectomy and then had relapsed metastatic disease 2 years after upfront treatment. Indeed, we did not find other DNA repair gene mutations in the 32‐gene genetic panel testing except for the known *BRCA*1 c.815_824dup10 (p.Thr276AlafsX14) mutation. Also, the tumor was negative for PD‐L1 expression (CPS 0) and the TIL score was zero. This could be due to the lack of assessment of the presence of homologous recombination deficiency (HRD) outside of the known germline gene mutations [37, 38] such as an HRD score tested in ovarian cancer [39, 40]. Further collaborative investigations beyond PD‐L1 expression and HRD status are needed to identify those exceptional responders.

In this study, we investigated pharmacodynamic and potential predictive biomarkers for response or lack of response to durvalumab and olaparib combination. As shown in KEYNOTE‐355, a PD‐L1 CPS positivity (CPS ≥ 10) was approved by the FDA as a biomarker to predict the response to pembrolizumab in combination with chemotherapy in locally recurrent unresectable or metastatic TNBC [3]. However, we did not find any association between clinical outcome measures and CPS. It is possible that negative PD‐L1 (CPS 0) in the two patients with durable PR may be related to the potential discordance between biopsied sites (i.e., breast tumor vs. metastatic site) [41]. Therefore, better spatial and temporal assessment of the PD‐L1 expression is warranted. Also, the higher number of stromal TILs as a continuous variable has been reported as a possible predictive biomarker for response to pembrolizumab in patients with metastatic TNBC [42, 43]. We observed no association between TILs and clinical outcomes although approximately half of tumors (7 of 13 evaluable patients) contained TILs, consistent with the previous report [44]. Multiple biopsies from the primary and metastatic sites [45] as well as serial biopsies would be informative to better understand the role of TILs and immunostimulatory effects of PARPi [13, 46], although it is logistically challenging.

It is well known that the immune cell profile in the TME is critically important to augment the efficacy of ICIs [10, 47]. However, because of the invasiveness of tissue biopsy, repeating biopsies is not feasible and practical. Therefore, the development of less invasive tests to predict the TME is an unmet clinical need. We investigated peripheral biomarkers as the use of circulating immune cells has been proposed to predict the immune profiles in TME [48, 49]. Among many other immune cell populations in peripheral blood, known to play a crucial role in remodeling the TME (e.g., myeloid derived suppressor cells, regulatory T cells [50, 51], memory T cells [52, 53], immune checkpoint and co‐stimulatory markers on T cells [54, 55, 56]), our findings exhibited lower CD83 expression of the DC maturation marker on both cDC1s and cDC2s at baseline in the benefit group (PFS ≥ 4 months) compared to the no benefit group (PFS < 4 months), which suggests that the circulating mature DCs may be associated with an immune suppressive TME as mature DCs negatively affect T cell immunity by inducing regulatory T cells (Tregs) as reported previously [57, 58]. Further detailed classification of DCs to understand the predictive role of DCs and prospective validation in a larger study are required.

There are several limitations in this study. First, this is a single‐arm single‐center pilot phase II trial. All efficacy and biomarker findings (e.g., immunohistochemistry) should be interpreted as only hypothesis‐generating due to the small sample size and no monotherapy arms. Also, the lack of serial biopsies and the nature of post hoc analysis provided only preliminary results requiring further investigation with a larger sample size. Second, genetic or somatic panel testing besides germline *BRCA* 1 and 2 was not mandated for enrollment. Therefore, there is a possibility that some patients with gBRCAwt might have had deleterious mutations in the homologous recombination repair pathway. Also, other correlative studies (e.g., transcriptomic analysis) would have provided more insights into tumor biology and treatment response.

In conclusion, our study demonstrated the modest clinical benefits (ORR of 28.6%) of the combination of durvalumab and olaparib in the patients with heavily pre‐treated TNBC with manageable adverse events. To further validate our findings, prospective clinical trials in a larger cohort are required. The proportions of activating type 1 and 2 cDCs in peripheral blood at baseline were found to be associated with PFS benefit. Further validation, mechanistic studies, and investigations for additional predictive biomarker candidates are warranted.

## Author Contributions

**Ashley Cimino‐Mathews:** data curation (equal), formal analysis (equal), investigation (equal), methodology (equal), resources (equal), software (equal), writing – review and editing (equal). **Ann McCoy:** data curation (equal), investigation (equal), project administration (equal), writing – review and editing (equal). **Bernadette Redd:** data curation (equal), methodology (equal), resources (equal), writing – review and editing (equal). **Britanny Brooke Solarz:** data curation (equal), investigation (equal), project administration (equal), writing – review and editing (equal). **Jayakumar Nair:** data curation (equal), formal analysis (equal), investigation (equal), methodology (equal), resources (equal), software (equal), writing – review and editing (equal). **Jung‐Min Lee:** conceptualization (lead), data curation (equal), formal analysis (equal), funding acquisition (lead), investigation (equal), methodology (lead), project administration (lead), resources (lead), software (equal), supervision (lead), writing – original draft (equal), writing – review and editing (equal). **Elliot B. Levy:** data curation (equal), investigation (equal), methodology (equal), resources (equal), writing – review and editing (equal). **Stanley Lipkowitz:** investigation (equal), methodology (equal), resources (equal), writing – review and editing (equal). **Min‐Jung Lee:** data curation (equal), formal analysis (equal), investigation (equal), methodology (equal), resources (equal), software (equal), writing – review and editing (equal). **Nahoko Sato:** formal analysis (equal), investigation (equal), methodology (equal), resources (equal), software (equal), writing – review and editing (equal). **Shraddha Rastogi:** data curation (equal), formal analysis (equal), investigation (equal), methodology (equal), resources (equal), software (equal), writing – review and editing (equal). **Seth M. Steinberg:** formal analysis (lead), writing – review and editing (equal). **Takeo Fujii:** data curation (lead), formal analysis (equal), investigation (equal), methodology (equal), software (equal), writing – original draft (lead), writing – review and editing (lead). **Alexandra Zimmer:** investigation (equal), methodology (equal), resources (equal), writing – review and editing (equal).

## Ethics Statement

The trial was approved by the Institutional Review Board (IRB) of the CCR, NCI. The study has been conducted in accordance with ethical principles that have their origin in the Declaration of Helsinki and are consistent with the International Council on Harmonization guidelines on Good Clinical Practice, all applicable laws and regulatory requirements, and all conditions required by a regulatory authority and/or IRB.

## Consent

All patients provided written informed consent.

## Conflicts of Interest

J.‐M.L. has research grant funding from AstraZeneca and Acrivon Therapeutics (paid to institution) and is on the Scientific Advisory Board of Acrivon Therapeutics (unpaid). The other authors declare no conflicts of interest.

## Supporting information


**Appendix S1:** cam471220‐sup‐0001‐AppendixS1.docx.

**Table S1:** Cell types and markers used for flow cytometry.
**Table S2:** Antibodies used in flow cytometry.
**Figure S1:** Dynamic changes of CTCs between baseline and C1D15 among no benefit and benefit groups.
**Figure S2:** Baseline between no benefit and benefit groups and dynamic changes between baseline and C1D15 in dendritic cells and clinical response.
**Figure S3:** Dynamic changes of non‐classical monocytes and plasmacytoid DC (pDC) between baseline and C1D15.
**Figure S4:** Dynamic changes between baseline and C1D15 in the systemic T cells.

## Data Availability

The datasets analyzed during the current study are available from the corresponding author and the senior author upon reasonable request.
