# Supplementary material for: A Phase II Pilot Study of Anti‐PD‐L1, Durvalumab, and a PARP Inhibitor, Olaparib in Patients With Metastatic Triple‐Negative Breast Cancer With or Without Germline BRCA Mutation
Source: Cancer Med. 2025 Dec 8;14(23):e71220. doi: 10.1002/cam4.71220 (PMC12685463; doi:10.1002/cam4.71220)
Supplement: Supplementary file 1 — Appendix S1: cam471220‐sup‐0001‐AppendixS1.docx. [file CAM4-14-e71220-s001.docx]

**PRINCIPAL INVESTIGATOR:** Jung-Min Lee, M.D.

**STUDY TITLE:** Phase I/II study of the anti-programmed death ligand-1 antibody Durvalumab (MEDI4736) in combination with olaparib and/or cediranib for advanced solid tumors and advanced or recurrent ovarian, triple negative breast, lung, prostate, and colorectal cancers

**STUDY SITE:** NIH Clinical Center

Cohort: Phase II Durvalumab+O Consent Version: September 9, 2021

# WHO DO YOU CONTACT ABOUT THIS STUDY?

Jung-Min Lee, MD, by phone at 240-760-6128 or email [leej6@mail.nih.gov](mailto:leej6@mail.nih.gov)

This consent form describes a research study and is designed to help you decide if you would like to be a part of the research study.

The remaining document will now describe the research study in more detail. This information should be considered before you make your choice. Members of the study team will talk with you about the information in this document. Some people have personal, religious, or ethical beliefs that may limit the kinds of medical or research interventions in which they would want to participate. Take the time you need to ask any questions and discuss this study with NIH staff, and with your family, friends, and personal health care providers.

If the individual being asked to participate in this research study is not able to give consent to be in this study, you are being asked to give permission for this person as their decision-maker. The term “you” refers to you as the decision-maker and/or the individual being asked to participate in this research, throughout the remainder of this document.

# IT IS YOUR CHOICE TO TAKE PART IN THE STUDY

You may choose not to take part in this study for any reason. If you join this study, you may change your mind and stop participating in the study at any time and for any reason. In either case, you will not lose any benefits to which you are otherwise entitled. However, to be seen at the NIH, you must be taking part in a study or are being considered for a study. If you do choose to leave the study, please inform your study team to ensure a safe withdrawal from the research.

# WHY IS THIS STUDY BEING DONE?

The purpose of the Phase I portion of this research study is to determine the safety of the combination of the drugs and the highest doses that can be given in combination to people safely. The resulting doses identified in the Phase I portion of the study will be used in the Phase II portion of the study. We will try to determine how effective each combination of the study medications is in shrinking tumors in patients with cancer. Two different combinations of drugs will be used:

1) Durvalumab in combination with cediranib (Durvalumab+C) or 2) Durvalumab in combination with olaparib (Durvalumab+O). In the Phase I study, a third combination using all three drugs will also be tested; i.e., Durvalumab in combination with olaparib and cediranib (Durvalumab+O+C). At this time there are no plans to test the three-drug combination in the Phase II study.

You are taking part in the Phase II Durvalumab+O portion of the study where it will be determined whether the drug combination shrinks your tumor. Durvalumab is a drug that may help your immune system respond to and kill your cancer cells. Olaparib is a drug that may stop cancer cells from growing abnormally, and is the first FDA approved PARP inhibitor for use in recurrent ovarian cancer with germline BRCA mutation. Durvalumab is investigational, meaning that they have not been approved by the FDA for use in patients. Also, combining Durvalumab with olaparib is investigational. These drugs have been used separately in other research studies in patients with solid tumors, including ovarian, breast, lung and prostate cancers. Information from those other research studies suggests that these may help to keep cancer from growing in this research study.

# WHY ARE YOU BEING ASKED TO TAKE PART IN THIS STUDY?

You are being asked to take part in this study because you have ovarian, fallopian tube, peritoneal, triple negative breast, lung, or prostate cancer that is persistent or has recurred.

# HOW MANY PEOPLE WILL TAKE PART IN THIS STUDY?

Up to 384 people will take part in this study, with up to 350 taking part in the phase II portion of the study.

# DESCRIPTION OF RESEARCH STUDY

**WHAT WILL HAPPEN IF YOU TAKE PART IN THIS RESEARCH STUDY?**

## Before you begin the study

You will need to undergo tests to determine if you are eligible to participate in the study. Most of the tests needed would be part of your routine medical care for your cancer. They include blood tests, physical examination, and CT scans. These tests will be performed on a separate protocol.

If it is determined that you are eligible for the study and you chose to sign the consent, you will be enrolled onto the study.

## During the study

Subjects enrolled in the Phase II portion of the study will be treated with the highest safe dose identified during the Phase I portion of the study.

## Medication

Durvalumab

You will be given the Durvalumab intravenously (IV) through a small plastic tube inserted into a vein (usually one on your arm). The drug will be given at a dose of 1500mg every 28 days. One cycle is 28 days. The drug will be given until your disease worsens or you experience an intolerable side effect.

Olaparib

You will take olaparib at a dose of 300mg in tablet form by mouth every day until either your disease worsens or you experience an intolerable side effect. Olaparib will be taken twice daily. You can take the olaparib with a light snack.

You will be given enough olaparib for 1 cycle (28 days). You should take the olaparib every day. You will be provided with a medication diary and a diarrhea diary. You will be asked to complete a drug diary stating when you took each dose or to give a reason if you did not take the study drug(s). Also, you will be asked to complete a diarrhea diary stating how many times you have a bowel movement so that doctors understand and prevent possible drug-related side effects better. At the end of each cycle, you will be asked to return the pill bottles and all of the remaining pills, and the two diaries prior to starting the next cycle.

If you have side effects that are difficult to manage or there are changes in your laboratory studies you may be asked to stop taking the study drug for a while or lower the number of the study tablet(s) you are taking.

Evaluations

In order for us to monitor any side effects you may have and to determine how well you are responding to the study medication, you will undergo the following tests/evaluations:

Unless otherwise indicated, the tests are performed on days 1 and 15 of cycles 1; then on day 1 of all subsequent cycles.

- Medical History (baseline/Day 1 only) For patients with breast or ovarian cancer, this includes documentation of whether or not they have a certain mutation associated with breast and ovarian cancer.
- Physical Examination including vital signs (Day 1 only for all cycles)
- Weight (performed on the day of Durvalumab infusion)
- Routine blood and urine tests
- CT-Scan or MRI (every 8 weeks)
- Electrocardiogram (EKG) before and after Durvalumab on Cycle 1 Day 1; before and after Durvalumab every 4 cycles, and at the end of study treatment
- Echocardiogram (ECHO) or multiple-gated acquisition (MUGA) scan at baseline and every 4 cycles thereafter for participants with certain conditions or prior treatment history. Your study team will tell you if this applies to you.
- If you have prostate cancer, CT-Scan or MRI and technetium-99 bone scan imaging initially at 8 weeks then every 12 weeks

Additional Research Evaluations

We will conduct various research studies that will help us determine how your body responds to and processes the study medications. The following samples will be collected for these purposes.

- To determine how your body responds to the medications, research blood samples will be collected before treatment on Cycle 1 Day 1 and Day 15, and at the end of Cycle 2. If your disease worsens, another sample will be collected at that time.
- We would like to collect a piece of your tumor (a biopsy) at three different times while you are on the study. The first biopsy will be done before you have begun any treatment on day 1 of cycle 1. This biopsy is required for you to participate in the study. The two additional biopsies are optional. One is collected at the end of cycle 1; and if your disease worsens, the other is collected at that time. You may indicate your choice to participate in the optional biopsies prior to each procedure.
- If you have prostate cancer, we would like to collect a one-time saliva sample as a source of normal tissue.

## When you are finished taking the drugs (treatment)

You will be asked to remain on study for at least 90 days after the final infusion of Durvalumab, or at least 30 days after the final dose of cediranib or olaparib, or until you receive subsequent cancer treatment, whichever occurs later. This is so that we may follow you for any side effects that are late in developing.

# BIRTH CONTROL

If you are a woman who is breast feeding or pregnant, you may not take part in the study because some of the medications used in this study may harm your unborn child. If you are a woman who can become pregnant, or are the partner of a woman who can become pregnant, you will need to practice two effective forms of birth control before starting study treatment, during study treatment, and for three months after you finish study treatment. If you think that you or your partner is pregnant, you should tell your study doctor or nurse at once.

Effective forms of birth control include:

- abstinence
- intrauterine device (IUD)
- hormonal [birth control pills, injections, or implants]
- tubal ligation
- vasectomy

## What tests will be done on my samples?

The analyses that we perform in our laboratory are for research purposes only; they are not nearly as sensitive as the tests that are performed in a laboratory that is certified to perform genetic testing. Changes that we observe unrelated to our research may or may not be valid. Therefore, we do not plan to inform you of the results of testing on your specimens that is performed in our research lab. However, in the unlikely event that we discover a finding that is believed to be clinically important based on medical standards at the time we first analyze your results, we will contact you. This could be many years in the future. We will ask you to have an additional tube of blood drawn to verify the findings we have seen in our lab. If the results are verified, you will be re- contacted and offered a referral to a genetic healthcare provider to discuss the results.

## Who else besides the investigators on this study will know the results of my sample testing?

Once we obtain any of the samples listed above, the investigators take all your personal information off those samples and label them with a study code number. Only the investigators on this study know from whom the sample came. The key linking your personal information with the code number is kept in a secure computer data base, with access only to the 2-3 research staff who will be discussing this study with you. Once the sample has been labeled with a code, it is sent to a variety of NIH laboratories for storage and testing. No one testing your samples will be able to link the results to you personally. Specimens obtained during your participation in this study may be sent for testing to investigators outside of NCI or the NIH. All samples will be coded to protect your privacy and no personal information will be included. Other investigators on this study will have access to limited clinical and biologic data such as age, gender and disease status.

## How long will your samples be stored?

The samples collected during this study will be stored for as long as the study is open. When this study is closed, we would like to keep the samples for future research.

# RISKS OR DISCOMFORTS OF PARTICIPATION

## General Risks

If you choose to take part in this study, there is a risk that:

- You may lose time at work or home and spend more time in the hospital or doctor’s office than usual
- You may be asked sensitive or private questions which you normally do not discuss

The drugs used in this study may affect how different parts of your body work such as your liver, kidneys, heart, and blood. The study doctor will be testing your blood and will let you know if changes occur that may affect your health.

There is also a risk that you could have side effects from the study drug(s)/study approach.

## Medication Risks

The study medications being administered in this study activate the immune system. The body’s immune system may react to drug treatment causing side effects with any organ system. Some of these potential side effects as noted in the risk sections below have been seen in clinical studies that have administered Durvalumab alone. Some of the side effects may occur during the infusions or after infusions (within hours to days after). The side effects usually get better without treatment. However, if left untreated, some may be serious or life-threatening.

Side effects that may be experienced as a result of the immune system’s reaction to medications that activate the immune system include the following:

- Fever
- Fatigue
- Rash or hives with or without itchiness and swelling
- Change in blood pressure sometimes significant
- Decrease in blood platelets (cells that stop bleeding) with symptoms such as unexpected bruising, bleeding from the nose or gums, blood in vomit or stools or red spots under the skin
- Inflammation of the lungs with symptoms including difficulty breathing
- Inflammation of the nervous system with symptoms including a tingling or burning feeling, sharp pain, weakness, numbness or reduced ability to feel pain and temperature changes especially in the fingers and toes and pain while walking
- Inflammation of the pancreas with symptoms such as abnormal laboratory blood tests (increases in amylase and lipase measure how your pancreas is working), stomach pain, nausea, vomiting and tenderness when touching the stomach
- Inflammation of the liver with symptoms such as stomach swelling, bloating, diarrhea, discolored urine or stools, loss of appetite, tiredness, nausea with or without vomiting, yellowing of the skin and whites of the eyes. Increases in the blood level of substances called enzymes found within your liver cells may occur. The enzyme changes are unlikely to make you feel unwell.
- Inflammation of the intestines with symptoms such as stomach pain, loose or more frequent stools, blood in stools which may require you to receive additional fluids. If left untreated this may lead to a tear in the wall of the intestine
- Symptoms related to changes in hormone-releasing glands including, but not limited to, increased heart rate, headaches, nausea, vomiting, fatigue, dizziness, weakness, tiredness, mood changes, loss of appetite, weight changes and sexual dysfunction
- Inflammation of the kidneys with symptoms such as abnormal blood tests, changes in urinary volume and pain in the abdomen
- Inflammation of the auditory system (ears) leading to symptoms such as sudden hearing loss in one or both ears. If left untreated this may lead to permanent hearing loss.
- Death

There is a small chance that you may have a serious allergic reaction to the study medications. Serious allergic reactions may occur during or after the infusion of study medication. The reaction may cause a serious change in blood pressure, difficulty in breathing, severe hives, and sometimes death. Your Study Doctor will monitor you very closely while you are receiving and after you receive study medication and will have medications available to treat any allergic reactions that might occur.

You may experience none, some or all of the side effects listed above or below. There may be risks involved in taking these drugs that have not been identified (unknown risks). There is always

a risk involved in taking a new drug but you will be closely monitored and you are encouraged to **immediately** report anything that is troubling you as **if left untreated, some of the side effects noted above and below could be life-threatening or fatal.** You will be told about any new findings that develop during the course of this study that may affect your decision to stay in the study.

Here are important points about side effects:

- The study doctors do not know who will or will not have side effects.
- Some side effects may go away soon, some may last a long time, or some may never go away.
- Some side effects may interfere with your ability to have children.
- Some side effects may be serious and may even result in death.

Here are important points about how you and the study doctor can make side effects less of a problem:

- You should talk to your study doctor about any symptoms that you experience while taking part in the study.
- The study doctor may be able to treat some side effects.
- The study doctor may adjust the study drugs to try to reduce side effects.

The tables below show the most common and the most serious side effects that researchers know about. There might be other side effects that researchers do not yet know about. If important new side effects are found, the study doctor will discuss these with you.

Risks of Durvalumab

Safety data from patients with various types of cancer who received Durvalumab alone is provided below.

**Related** side effects reported in subjects receiving Durvalumab alone were:

- Fatigue
- Difficulty breathing
- Nausea
- Constipation
- Decreased appetite
- Diarrhea
- Vomiting
- Rash/dry itchy skin
- Increased liver enzymes
- Cough

**LIKELY, SOME MAY BE SERIOUS**

In 100 people receiving Durvalumab, more than 10 and up to 25 may have:

- Fever
- Pain in muscles and joints
- Hypothyroidism is an underactive thyroid (when it produces too little thyroid hormone), which can cause constipation, fatigue, sensitivity to cold, weight gain, dry skin, and/or forgetfulness.

# LESS LIKELY, SOME MAY BE SERIOUS

In 100 people receiving Durvalumab, from 2 to fewer than 10 may have:

- - Inflammation in the lungs (pneumonitis that can be fatal)
  - Sensitivity to cold
  - Unexplained loss or gain in weight
  - Puffy face
  - Slow heart rate
  - Thinning hair
  - Impaired memory
  - Anxiety or nervousness
  - Feeling hot and possibly having heart palpitations.
  - Decreased kidney function
  - Weakness of legs, arms, or face, numbness or tingling in hands or feet
  - During or after drug infusion having:
    - fever
    - chills
    - change in blood pressure
    - difficulty in breathing which might be serious
  - Inflammation of the colon which can lead to abdominal pain and diarrhea with or without blood. If left untreated, this may lead to a tear in the wall of the intestine which can be serious and life threatening.
  - Hyperthyroidism is an overactive thyroid (when it produces too much thyroid hormone), which can cause tremors, weight loss, nervousness, fast heart rate, trouble sleeping, brittle skin, muscle weakness, irritability, and/or lighter or less frequent menstrual periods.

# RARE, SOME MAY BE SERIOUS

In 100 people receiving Durvalumab, 2 or fewer may have:

- - Depression, changes in mood and personality
  - Change in blood pressure
  - Increased pancreas enzymes: lipase and amylase.
  - Type 1 Diabetes mellitus (high blood sugar)
  - Allergic reactions, causing:
    - swelling of the face, lips and throat
    - breathing difficulties which might be serious
    - hives or nettle like rash
    - change in blood pressure
  - Headaches
  - Thirstiness
  - Trouble seeing or double vision
  - Leakage of breast milk or irregular periods in women.
  - Inflammation of the liver called hepatitis, that can be fatal
  - Inflammation (swelling and redness) of the eyes, ranging from mild to moderate
  - Inflammation (swelling and redness) of the joints (arthritis), ranging from mild to moderate
  - Inflammation of the heart, that can be fatal
  - Inflammation of the blood vessels, that can be fatal
  - Inflammation of the brain, that can be fatal
  - Problems swallowing
  - New allergies to previously exposed substances, other than Durvalumab. For example, it is possible that you could develop an allergy to shellfish or IV contrast while taking Durvalumab. These allergies may be severe and life threatening
  - Hearing loss
  - Neoplasm including malignant or benign, such as polyps or cysts
  - Rare skin conditions such as pemphigoid (blistering and rashes), vitiligo (loss of skin color in patches) and scleroderma (hardening and tightening of the skin)
  - Weakness and tingling of legs and arms called Guillain-Barre Syndrome, a rare disorder in which your body's immune system attacks your nerves
  - Muscle weakness affecting the eyes, face, and swallowing called Myasthenia Gravis
  - the growth of tiny collections of inflammatory cells (granulomas) in any part of your body called sarcoidosis
  - Anemia or low platelets counts, caused by the destruction of red blood cells or platelets
  - Hypophysitis is inflammation of the pituitary gland, a kidney-bean-sized gland situated at the base of your brain. The most common symptom is a headache which becomes severe with no relief. There may be nausea and vomiting.
  - Hypopituitarism is a condition where your pituitary gland fails to produce one or more hormones, or doesn't produce enough hormones. Symptoms may include stomach pain, decreased appetite, nausea, vomiting, constipation, excessive thirst and urination, fatigue, weakness, anemia, headache, dizziness, sensitivity to cold, weight loss or weight gain, and/or muscles aches.
  - Diabetes insipidus can be caused by the above two disorders and is not related to diabetes mellitus, but it shares some of the same symptoms, mainly extreme thirst and frequent urination.
  - Thyroiditis, which may exhibit symptoms such as weight loss, anxiety, irritability, trouble sleeping, rapid heart rate, fatigue, muscle weakness, and/or tremors (shaking hands or fingers).
  - Adrenal insufficiency, which may include fatigue, muscle weakness, loss of appetite, weight loss, and/or abdominal pain.

These symptoms might or might not be caused by problems with your liver, kidneys, colon, pancreas, nervous system, thyroid, adrenal or pituitary gland, affected by Durvalumab.

Tell your study doctor right away if you have any of these symptoms as they may need to be treated urgently.

A new drug may show an increase in side effects or unexpected effects as more studies are conducted. For your safety, you will be followed closely by your Study Doctor and the study staff for any undesirable or unexpected side effects during your participation in this study and each time you receive Durvalumab.

There may be other side effects of Durvalumab that are unknown. You will be told about any new findings that develop during the course of this study that may affect your decision to stay in the study.

# PATIENT IDENTIFICATION


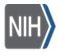


## Consent to Participate in a Clinical Research Study

NIH-2977 (4-17)

File in Section 4: Protocol Consent (#4)

Version Date: 09/09/2021 Page **10** of **22**

IRB NUMBER: 15C0145

IRB APPROVAL DATE: 09/15/2021

Risks of Olaparib

# COMMON, SOME MAY BE SERIOUS

In 100 people receiving olaparib, more than 10 and up to 100 may have:

- Low red blood cell count (anemia) which may cause you to feel tired, weak, or have shortness of breath. In some instance, you may require blood transfusions (when you are given new blood or blood products from a donor).
- Low white blood cell count, which can sometimes lead to infection. This does not mean you will get an infection, but it is important that you contact us immediately if you have a fever. This may require treatment with antibiotics.
- Decreased number of lymphocytes, a type of white blood cells. This is associated with an increased risk of infection.
- Nausea or vomiting (feeling sick or actually being sick). If required, you will be offered medication to control these symptoms.
- Fatigue
- Diarrhea, which is frequent, loose watery stools, which can cause dehydration and may require hospitalization and treatment with intravenous fluids. Severe and prolonged diarrhea can be life-threatening.
- Heartburn (dyspepsia)
- Pain in the upper part of your abdomen (upper abdominal pain)
- Decreased appetite
- Headache (pain in the head)
- Altered taste
- Dizziness
- Increase in blood creatinine, a substance normally eliminated by the kidneys into the urine. This may mean that your kidneys are not functioning properly.
- Low number of platelets, which may cause bleeding and bruising. Bleeding may be serious or life threatening and may require a blood transfusion.

**OCCASIONAL, SOME MAY BE SERIOUS**

In 100 people receiving olaparib, from 1 to 10 may have:

## Consent to Participate in a Clinical Research Study

|  | **RARE, AND SERIOUS**  In 100 people receiving olaparib, fewer than 1 may have: | |
| --- | --- | --- |
|  | - Inflammation of the lung tissue (pneumonitis), which may cause new or worsening symptoms of shortness of breath. This may be serious or life threatening. - Myelodysplasia (irreversible abnormal blood counts and bone marrow damage, which may lead to leukemia) - Myelodysplastic syndrome, a group of bone marrow disorders where the body no longer makes enough normal blood cells in the bone marrow, which may lead to leukemia | |
| **PATIENT IDENTIFICATION** | |  |

NIH-2977 (4-17)


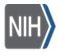
File in Section 4: Protocol Consent (#4)

Version Date: 09/09/2021 Page **11** of **22**

IRB NUMBER: 15C0145

IRB APPROVAL DATE: 09/15/2021

- Normocytic anemia, a condition where the red blood cells are normal in shape and size, but there are not enough circulating red blood cells to meet your body's needs
- Refractory anemia with an excess of blasts, a type of anemia with increased immature blood cells in the bone marrow and/or peripheral blood that is unresponsive to treatment
- Myeloid leukemia, a type of cancer in which the bone marrow makes abnormal myeloblasts (a type of white blood cell), red blood cells, or platelets
- Acute myeloid leukemia (AML), a fast-growing disease in which too many immature white blood cells are found in the bone marrow and blood
- Acute erythroid leukemia, a rare form of acute myeloid leukemia (less than 5% of AML cases) with a rapid increase in immature red blood cells
- Chronic myelomonocytic leukemia. a type of chronic blood cancer in which a person's bone marrow does not make blood effectively
- Angioedema, rapid swelling beneath the skin or mucosa
- Erythema nodosum, tender bumps (nodules) under the skin, which can cause pain
- Rash macular, a rash in which the lesions are flat and level with the surrounding skin
- New primary malignancies have been reported in a small number of people taking olaparib

**RARE, AND SERIOUS**

In 100 people receiving olaparib, fewer than 1 may have:

Other effects that have been reported by other subjects taking olaparib, although it is not clear that they are related to olaparib, include:

- Constipation
- Abdominal pain
- Fever– an elevated body temperature
- Swelling in the legs or arms (peripheral edema)
- A fast heartbeat (tachycardia)
- Changes in your thinking, including increased difficulty paying attention or troubles with your memory
- Sleepiness
- Depression or feeling sad **Research Procedure Risks Blood Draw**

There is the risk of slight pain, bruising or infection when your blood is drawn. Drawing blood may *cause* some people to faint.

## Biopsy

Common side effects of a biopsy are a small amount of bleeding at the time of the procedure, pain at the biopsy site, which can be treated with regular pain medications, and bruising. Rarely, an infection can occur

## Radiation risks

### *If you are in Cohorts 1 (ovarian cancer), 2 (NSCLC), 3 (SCLC), or 5 (breast cancer):*

During your participation in this research study, you will be exposed to radiation from CT scans. The amount of radiation exposure you will receive from these procedures is equal to approximately

11.2 rem. A rem is a unit of absorbed radiation.

Every day, people are exposed to low levels of radiation that come from the sun and the environment around them. The average person in the United States receives a radiation exposure of 0.3 rem per year from these sources. This type of radiation is called “background radiation”. This study will expose you to more radiation than you get from everyday background radiation. No one knows for sure whether exposure to these low amounts of radiation is harmful to your body.

The CT scans that you get in this study will expose you to roughly the same amount of radiation as 37.3 years’ worth of background radiation. Being exposed to too much radiation can cause harmful side effects such as an increase in the risk of cancer. The risk depends on how much radiation you are exposed to. Please be aware that about 40 out of 100 people (40%) will get cancer during their lifetime, and 20 out of 100 (20%) will die from cancer. The risk of getting cancer from the radiation exposure in this study is 1.1 out of 100 (1.1%) and of getting a fatal cancer is 0.6 out of 100 (0.6%).

You may not participate in this study if you are pregnant. If you are able to become pregnant, we will perform a pregnancy test before exposing you to radiation. You must tell us if you may have become pregnant within the previous 14 days because the pregnancy test is unreliable during that time.

### *If you are in Cohort 4 (prostate cancer):*

During your participation in this research study, you will be exposed to radiation from CT scans and Technetium-99 bone scans. The amount of radiation exposure you will receive from these procedures is equal to approximately 11.34 rem. A rem is a unit of absorbed radiation.

Every day, people are exposed to low levels of radiation that come from the sun and the environment around them. The average person in the United States receives a radiation exposure of 0.3 rem per year from these sources. This type of radiation is called “background radiation”. This study will expose you to more radiation than you get from everyday background radiation. No one knows for sure whether exposure to these low amounts of radiation is harmful to your body.

The CT scans that you get in this study will expose you to roughly the same amount of radiation as 37.8 years’ worth of background radiation. Being exposed to too much radiation can cause harmful side effects such as an increase in the risk of cancer. The risk depends on how much radiation you are exposed to. Please be aware that about 40 out of 100 people (40%) will get cancer

during their lifetime, and 20 out of 100 (20%) will die from cancer. The risk of getting cancer from the radiation exposure in this study is 1.1 out of 100 (1.1%) and of getting a fatal cancer is 0.6 out of 100 (0.6%).

You may not participate in this study if you are pregnant. If you are able to become pregnant, we will perform a pregnancy test before exposing you to radiation. You must tell us if you may have become pregnant within the previous 14 days because the pregnancy test is unreliable during that time.

## Risks of CT Scans

A CT scan is an X-ray procedure where a high-speed computer is used to make multiple images or pictures of your body. Sometimes, a contrast dye that contains iodine is administered into one of your veins to improve these images. There is a chance of developing an allergic reaction from the contrast material, which may cause symptoms ranging from mild itching or a rash to severe difficulty breathing, shock or rarely, death. The contrast material may also cause kidney problems. The study doctors will do a blood test prior to the test to confirm that it is safe you to receive the contrast. You may feel discomfort when the contrast material is injected. You may feel warm, flushed, get a metallic taste in your mouth or, rarely, may make you vomit or feel sick to your stomach. You will be asked to lie still on a table and at times may have to hold your breath for a few seconds in order to avoid blurring the pictures. You may hear a slight buzzing, clicking and/or whirring sounds as the CT scanner moves around your body.

*MRI scans*

People are at risk for injury from the MRI magnet if they have some kinds of metal in their body. It may be unsafe for you to have an MRI scan if you have pacemakers or other implanted electrical devices, brain stimulators, some types of dental implants, aneurysm clips (metal clips on the wall of a large artery), metal prostheses (including metal pins and rods, heart valves, and cochlear implants), permanent eyeliner, tattoos, an implanted delivery pump, or shrapnel fragments. Welders and metal workers may have small metal fragments in the eye. You will be screened for these conditions before having any MRI scan. If you have a question about metal in your body, you should inform the staff. You will be asked to complete an MRI screening form before each MRI scan you have.

In addition, all magnetic objects (like watches, coins, jewelry, and credit cards) must be removed before entering the MRI scan room.

People with fear of confined spaces may become anxious during an MRI. Those with back problems may have back pain or discomfort from lying in the scanner. The noise from the scanner is loud enough to damage hearing, especially in people who already have hearing loss. Everyone having a research MRI scan will be fitted with hearing protection. If the hearing protection comes loose during the scan, you should let us know right away.

There are no known long-term risks of MRI scans.

During part of the MRI you will receive gadolinium, a contrast agent, through an intravenous (IV) catheter (small tube). It will be done for research purposes.

It is not known if MRI with contrast is completely safe for a developing fetus. Therefore, all women of childbearing potential will have a pregnancy test performed no more than 24 hours before each MRI scan with contrast. The scan will not be done if the pregnancy test is positive.

The risks of an IV catheter include bleeding, infection, or inflammation of the skin and vein with pain and swelling.

Mild symptoms from gadolinium infusion occur in fewer than 1% of those who receive it and usually go away quickly. Mild symptoms may include coldness in the arm during the injection, a metallic taste, headache, and nausea. In an extremely small number, fewer than one in 300,000 people, more severe symptoms have been reported including shortness of breath, wheezing, hives, and lowering of blood pressure. You should not receive gadolinium if you previously had an allergic reaction to it. You will be asked about such allergic reactions before gadolinium is given.

People with kidney disease are at risk for a serious reaction to gadolinium contrast called “nephrogenic systemic fibrosis (NSF)”. This condition always involves the skin and can also involve the muscles, joints and internal organs. NSF has resulted in a very small number of deaths. A blood test of your kidney function may be done within the month before an MRI scan with gadolinium contrast. You will not receive gadolinium for a research MRI scan if your kidney function is below the safe level.

Most of the gadolinium contrast leaves the body in the urine. However, the FDA has issued a safety alert that indicates small amounts of gadolinium may remain in the body for months to years. The effects of the retained gadolinium are not clear. At this time, retained gadolinium has not been linked to health risks in people whose kidneys work well. Some types of gadolinium contrast drugs are less likely to remain in the body than others. In this study, we will use the gadolinium contrast drugs that are less likely to remain in the body, whenever possible. We will also give you additional information called a “Medication Guide.” Upon request, we will give you individual information about retained gadolinium we see on your studies.

## Risks of Technetium-99 Bone Scans - Cohort 4 (prostate cancer) only

This scan is a procedure that uses Technetium 99m to trace cancer that has spread (metastasized) to the bone from the tumor's original location; in this case, from the prostate. In addition to the risks of radiation described above, this test requires administration of the contrast agent. You may experience pain, bruising, and/or infection at the site of injection, or an allergic reaction to the contrast agent.

### *Psychological or Social Risks Associated with Return of Incidental or Secondary Findings*

As part of the research study, it is possible that you could learn that you have genetic risks for another disease or disability. This may be upsetting and, depending on what you learn, might create a need to make challenging decisions about how to respond.

Although your genomic information is unique to you, you share some genomic similarities with your children, parents, brothers, sisters, and other blood relatives. Therefore, learning your research results could mean something about your family members and might cause you or your family distress. Before joining the study, it may be beneficial to talk with your family members about whether and how they want you to share your results with them.

### *Privacy Risks Associated with Return of Incidental or Secondary Findings*

Your privacy is very important to us and we will use many safety measures to protect your privacy. However, in spite of all of the safety measures that we will use, we cannot guarantee that your identity will never become known. While neither the public nor the controlled-access databases developed for this project will have information such as your name, address, telephone number, or social security number, it may be possible to identify you based on the information in these databases and other public information (including information you tell people or post about yourself). The risk of this happening is currently very low.

Although your genetic information is unique to you, you do share some genetic information with your children, parents, brothers, sisters, and other blood relatives. Consequently, it may be possible that genetic information from them could be used to help identify you. Similarly, it may be possible that genetic information from you could be used to help identify them. Patterns of genetic variation also can be used by law enforcement agencies to identify a person or his/her blood relatives.

It is possible also that someone could get unauthorized access or break into the system that stores information about you. Every precaution will be taken to minimize this risk. There also may be other privacy risks that we have not foreseen.

### *Protections against misuse of genetic information*

This study involves genetic testing on samples. Some genetic information can help predict future health problems of you and your family and this information might be of interest to your employers or insurers. The Genetic Information Nondiscrimination Act (GINA) is a federal law that prohibits plans and health insurers from requesting genetic information or using genetic information. It also prohibits employment discrimination based on your health information. However, GINA does not address discrimination by companies that sell life insurance, disability insurance, or long-term care insurance. GINA also does not protect you against discrimination based on an already-diagnosed condition or disease that has a genetic component.

# POTENTIAL BENEFITS OF PARTICIPATION

## Are there benefits to taking part in this study?

The aim of this study is to determine the highest safe dose of this experimental treatment and to determine if it will cause your tumors to shrink. We do not know if you will receive personal, medical benefit from taking part in this study. These potential benefits could include shrinking of your tumor or lessening of your symptoms, such as pain, that are caused by the cancer. Because there is not much information about the drug s’ effect on your cancer, we do not know if you will benefit from taking part in this study, although the knowledge gained from this study may help others in the future who have cancer.

# ALTERNATIVE APPROACHES OR TREATMENTS

**WHAT OTHER CHOICES DO I HAVE IF I DO NOT TAKE PART IN THIS STUDY?**

Instead of being in this study, you have these options**:**

- Getting treatment or care for your cancer without being in a study
- Taking part in another study
- Getting comfort care, also called palliative care. This type of care helps reduce pain, tiredness, appetite problems and other problems caused by the cancer. It does not treat the cancer directly. Instead, it tries to improve how you feel. Comfort care tries to keep you as active and comfortable as possible.

Please talk to your doctor about these and other options.

# STOPPING THERAPY

Your doctor may decide to stop your therapy for the following reasons:

- if he/she believes that it is in your best interest
- if your disease comes back during treatment
- if you have side effects from the treatment that your doctor thinks are too severe
- if you become pregnant
- if new information shows that another treatment would be better for you In this case, you will be informed of the reason therapy is being stopped.

You can stop taking part in the study at any time. However, if you decide to stop taking part in the study, we would like you to talk to the study doctor and your regular doctor first.

If you decide at any time to withdraw your consent to participate in the trial, we will not collect any additional medical information about you. However, according to FDA guidelines, information collected on you up to that point may still be provided to MedImmune and AstraZeneca or designated representatives. If you withdraw your consent and leave the trial, any samples of yours that have been obtained for the study and stored at the NCI can be destroyed upon request. However, any samples and data generated from the samples that have already been distributed to other researchers or placed in the research databases can**not** be recalled and destroyed.

# CONFLICT OF INTEREST

The National Institutes of Health (NIH) reviews NIH staff researchers at least yearly for conflicts of interest. This process is detailed in a Protocol Review Guide. You may ask your research team for a copy of the Protocol Review Guide or for more information. Members of the research team who do not work for NIH are expected to follow these guidelines but they do not need to report their personal finances to the NIH.

Members of the research team working on this study may have up to $15,000 of stock in the companies that make products used in this study. This is allowed under federal rules and is not a conflict of interest.

The National Institutes of Health and the research team for this study are using drugs developed by MedImmune and AstraZeneca through a joint study with your researchers and the company. The company also provides financial support for this study.

# USE OF SPECIMENS AND DATA FOR FUTURE RESEARCH

To advance science, it is helpful for researchers to share information they get from studying human samples. They do this by putting it into one or more scientific databases, where it is stored along with information from other studies. A researcher who wants to study the information must apply to the database and be approved. Researchers use specimens and data stored in scientific databases to advance science and learn about health and disease.

We plan to keep some of your specimens and data that we collect and use them for future research and share them with other researchers. We will not contact you to ask about each of these future uses. These specimens and data will be stripped of identifiers such as name, address or account number, so that it may be used for future research on any topic and shared broadly for research purposes. Your specimens and data will be used for research purposes only and will not benefit you. It is also possible that the stored specimens and data may never be used. Results of research done on your specimens and data will not be available to you or your doctor. It might help people who have cancer and other diseases in the future.

If you do not want your stored specimens and data used for future research, please contact us in writing and let us know that you do not want us to use your specimens and/or data. Then any specimens that have not already been used or shared will be destroyed and your data will not be used for future research. However, it may not be possible to withdraw or delete materials or data once they have been shared with other researchers.

## Genomic Data Sharing

As part of this research study, we will put your genomic data in a large database for broad sharing with the research community. These databases are commonly called data repositories. The information in this database will include but is not limited to genetic information, race and ethnicity, and sex. If your individual data are placed in one of these repositories*,* they will be labeled with a code and not with your name or other information that could be used to easily identify you, and only qualified researchers will be able to access them. These researchers must receive prior approval from individuals or committees with authority to determine whether these researchers can access the data.

Summary information about all of the participants included in this study (including you*)* is being placed in a database and will be available through open access. That means that researchers and non-researchers will be able to access summary information about all the participants included in the study, or summary information combined from multiple studies, without applying for permission. The risk of anyone identifying you with this information is very low.

NIH policies require that genomic data be placed in a repository for sharing. Therefore, we cannot offer you a choice of whether your data will be shared. If you do not wish to have your data placed in a repository, you should not enroll in this study.

# COMPENSATION, REIMBURSEMENT, AND PAYMENT

## Will you receive compensation for participation in the study?

Some NIH Clinical Center studies offer compensation for participation in research. The amount of compensation, if any, is guided by NIH policies and guidelines.

You will not receive compensation for participation in this study.

## Will you receive reimbursement or direct payment by NIH as part of your participation?

Some NIH Clinical Center studies offer reimbursement or payment for travel, lodging or meals while participating in the research. The amount, if any, is guided by NIH policies and guidelines.

On this study, the NCI will cover the cost for some of your expenses. Some of these costs may be paid directly by the NIH and some may be reimbursed after you have paid. Someone will work with you to provide more information.

## Will taking part in this research study cost you anything?

NIH does not bill health insurance companies or participants for any research or related clinical care that you receive at the NIH Clinical Center.

- - If some tests and procedures are performed outside the NIH Clinical Center, you may have to pay for these costs if they are not covered by your insurance company.
  - Medicines that are not part of the study treatment will not be provided or paid for by the NIH Clinical Center.
  - Once you have completed taking part in the study, medical care will no longer be provided by the NIH Clinical Center.

# CLINICAL TRIAL REGISTRATION AND RESULTS REPORTING

A description of this clinical trial will be available on [http://www.ClinicalTrials.gov](http://www.ClinicalTrials.gov/), as required by U.S. Law. This Web site will not include information that can identify you. At most, the Web site will include a summary of the results. You can search this Web site at any time.

# CONFIDENTIALITY PROTECTIONS PROVIDED IN THIS STUDY

## Will your medical information be kept private?

We will do our best to make sure that the personal information in your medical record will be kept private. However, we cannot guarantee total privacy. Organizations that may look at and/or copy your medical records for research, quality assurance, and data analysis include:

- - The NIH and other government agencies, like the Food and Drug Administration (FDA), which are involved in keeping research safe for people.
  - National Institutes of Health Intramural Institutional Review Board
  - The study sponsor, National Cancer Institute Center for Cancer Research, or their agent(s)
  - Qualified representatives from MedImmune, the pharmaceutical company who produces Durvalumab and AstraZeneca, the pharmaceutical company who produces olaparib and cediranib.

When results of an NIH research study are reported in medical journals or at scientific meetings, the people who take part are not named and identified. In most cases, the NIH will not release any information about your research involvement without your written permission. However, if you sign a release of information form, for example, for an insurance company, the NIH will give the insurance company information from your medical record. This information might affect (either favorably or unfavorably) the willingness of the insurance company to sell you insurance.

If we share your specimens or data with other researchers, in most circumstances we will remove your identifiers before sharing your specimens or data. You should be aware that there is a slight possibility that someone could figure out the information is about you.

Further, the information collected for this study is protected by NIH under a Certificate of Confidentiality and the Privacy Act.

## Certificate of Confidentiality

To help us protect your privacy, the NIH Intramural Program has received a Certificate of Confidentiality (Certificate). With this certificate, researchers may not release or use data or information about you except in certain circumstances.

NIH researchers must not share information that may identify you in any federal, state, or local civil, criminal, administrative, legislative, or other proceedings, for example, if requested by a court.

The Certificate does not protect your information when it:

1. is disclosed to people connected with the research, for example, information may be used for auditing or program evaluation internally by the NIH; or
2. is required to be disclosed by Federal, State, or local laws, for example, when information must be disclosed to meet the legal requirements of the federal Food and Drug Administration (FDA);
3. is for other research;
4. is disclosed with your consent.

The Certificate does not prevent you from voluntarily releasing information about yourself or your involvement in this research.

The Certificate will not be used to prevent disclosure to state or local authorities of harm to self or others including, for example, child abuse and neglect, and by signing below you consent to those disclosures. Other permissions for release may be made by signing NIH forms, such as the Notice and Acknowledgement of Information Practices consent.

## Privacy Act

The Federal Privacy Act generally protects the confidentiality of your NIH medical records we collect under the authority of the Public Health Service Act. In some cases, the Privacy Act protections differ from the Certificate of Confidentiality. For example, sometimes the Privacy Act allows release of information from your medical record without your permission, for example, if it is requested by Congress. Information may also be released for certain research purposes with due consideration and protection, to those engaged by the agency for research purposes, to certain federal and state agencies, for HIV partner notification, for infectious disease or abuse or neglect

reporting, to tumor registries, for quality assessment and medical audits, or when the NIH is involved in a lawsuit. However, NIH will only release information from your medical record if it is permitted by both the Certificate of Confidentiality and the Privacy Act.

# POLICY REGARDING RESEARCH-RELATED INJURIES

The NIH Clinical Center will provide short-term medical care for any injury resulting from your participation in research here. In general, no long-term medical care or financial compensation for research-related injuries will be provided by the NIH, the NIH Clinical Center, or the Federal Government. However, you have the right to pursue legal remedy if you believe that your injury justifies such action.

# PROBLEMS OR QUESTIONS

If you have any problems or questions about this study, or about your rights as a research participant, or about any research-related injury, contact the Principal Investigator, Jung-min Lee, M.D., [leej6@mail.nih.gov](mailto:leej6@mail.nih.gov), 240-760-6128. You may also call the NIH Clinical Center Patient Representative at 301-496-2626, or the NIH Office of IRB Operations at 301-402-3713, if you have a research-related complaint or concern.

# CONSENT DOCUMENT

Please keep a copy of this document in case you want to read it again.

**Adult Research Participant:** I have read the explanation about this study and have been given the opportunity to discuss it and to ask questions. I consent to participate in this study.

Signature of Research Participant Print Name of Research Participant Date

**Legally Authorized Representative (LAR) for an Adult Unable to Consent:** I have read the explanation about this study and have been given the opportunity to discuss it and to ask questions. I am legally authorized to make research decisions on behalf of the adult participant unable to consent and have the authority to provide consent to this study. As applicable, the information in the above consent was described to the adult participant unable to consent who agrees to participate in the study.

| Signature of LAR |  | Print Name of LAR |  | Date |
| --- | --- | --- | --- | --- |
| **Investigator:** |  |  |  |  |
| Signature of Investigator |  | Print Name of Investigator |  | Date |

## Witness to the oral short-form consent process only:

**Witness:**

Signature of Witness***** Print Name of Witness Date

# *NIH ADMINISTRATIVE SECTION TO BE COMPLETED REGARDING THE USE OF AN INTERPRETER:

An interpreter, or other individual, who speaks English and the participant’s preferred language facilitated the administration of informed consent and served as a witness. The investigator obtaining consent may not also serve as the witness.

An interpreter, or other individual, who speaks English and the participant’s preferred language facilitated the administration of informed consent but did not serve as a witness. The name or ID code of the person providing interpretive support is: .

# PATIENT IDENTIFICATION


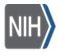


## Consent to Participate in a Clinical Research Study

NIH-2977 (4-17)

File in Section 4: Protocol Consent (#4)

Version Date: 09/09/2021 Page **22** of **22**

IRB NUMBER: 15C0145

IRB APPROVAL DATE: 09/15/2021
