# Supplementary material for: A Phase II Pilot Study of Anti‐PD‐L1, Durvalumab, and a PARP Inhibitor, Olaparib in Patients With Metastatic Triple‐Negative Breast Cancer With or Without Germline BRCA Mutation
Source: Cancer Med. 2025 Dec 8;14(23):e71220. doi: 10.1002/cam4.71220 (PMC12685463; doi:10.1002/cam4.71220)
Supplement: Supplementary file 2 — Table S1: Cell types and markers used for flow cytometry. Table S2: Antibodies used in flow cytometry. Figure S1: Dynamic changes of CTCs between baseline and C1D15 among no benefit and benefit groups. Figure S2: Baseline between no benefit and benefit groups and dynamic changes between baseline and C1D15 in dendritic cells and clinical response. Figure S3: Dynamic changes of non‐classical monocytes and plasmacytoid DC (pDC) between baseline and C1D15. Figure S4: Dynamic changes between baseline and C1D15 in the systemic T cells. [file CAM4-14-e71220-s002.docx]

Supplementary information

**Table of Contents**

**Table S1.** Cell types and markers used for flow cytometry **2**

**Table S2.** Antibodies used in flow cytometry**3**

**Figure S1.** Dynamic changes of CTCs between baseline and C1D15 among no benefit and benefit groups**4**

**Figure S2.** Baseline between no benefit and benefit groups and dynamic changes between baseline and C1D15 in dendritic cells and clinical response **5**

**Figure S3.** Dynamic changes of non-classical monocytes and plasmacytoid DC (pDC) between baseline and C1D15**6**

**Figure S4.** Dynamic changes between baseline and C1D15 in the systemic T cells **7**

**Data S1.** Inclusion and Exclusion criteria **8**

**Data S2.** Dosing delays/dose modifications and management of toxicities**11**

|  |  |  |  |
| --- | --- | --- | --- |
|  | Table S1. Cell types and markers used for flow cytometry | | |
|  | **Cell types** |  | **Markers** |
|  | Non-classical monocytes |  | CD14 dim CD16+ |
|  | Effector T cells |  | CD45RA- CCR7- CD28- CD27- (CD4+ or CD8+) |
|  | Activated proliferating T cells |  | (HLA-DR+ or ICOS+) Ki-67+ (CD4+ or CD8+) |
|  | cDC1s |  | Lin (CD3, CD19, CD56)- HLA-DR+ CD11c+ CD141+ |
|  | cDC2s |  | Lin (CD3, CD19, CD56)- HLA-DR+ CD11c+ CD1c+ |
|  | pDCs |  | Lin (CD3, CD19, CD56)- HLA-DR+ CD11c+ CD303+ |
|  | Abbreviations: HLA-DR, human leukocyte antigen-DR isoform; ICOS, inducible T cell co-stimulator; cDC1s, type 1 conventional dendritic cells; cDC2s, type 2 DCs; Lin, lineage; pDCs, plasmacytoid DCs | | |
|  |  |  |  |

|  |  |  |  |  |  |
| --- | --- | --- | --- | --- | --- |
|  | Table S2. Antibodies used in flow cytometry | | | | |
|  | **Surface marker** |  | **Clone** |  | **Company** |
|  | CD14 |  | HCD14 |  | BioLegend |
|  | HLA-DR |  | LN3 |  | BioLegend |
|  | CD16 |  | 3G8 |  | BioLegend |
|  | CD3 |  | OKT3 |  | BioLegend |
|  | CD4 |  | RPAT4 |  | BioLegend |
|  | CD8 |  | SK1 |  | BioLegend |
|  | CD45RA |  | HI100 |  | BioLegend |
|  | ICOS |  | C398.4A |  | BioLegend |
|  | Ki-67 |  | B56 |  | BD Biosciences |
|  | CD19, |  | HIB19 |  | BioLegend |
|  | CD56 |  | MEM-188 |  | BioLegend |
|  | CD11c |  | Bu15 |  | BioLegend |
|  | CD141 |  | M80 |  | BioLegend |
|  | CD1c |  | L161 |  | BioLegend |
|  | CD303 |  | 201A |  | BioLegend |
|  | PD-1 |  | EH12.2H7 |  | BioLegend |


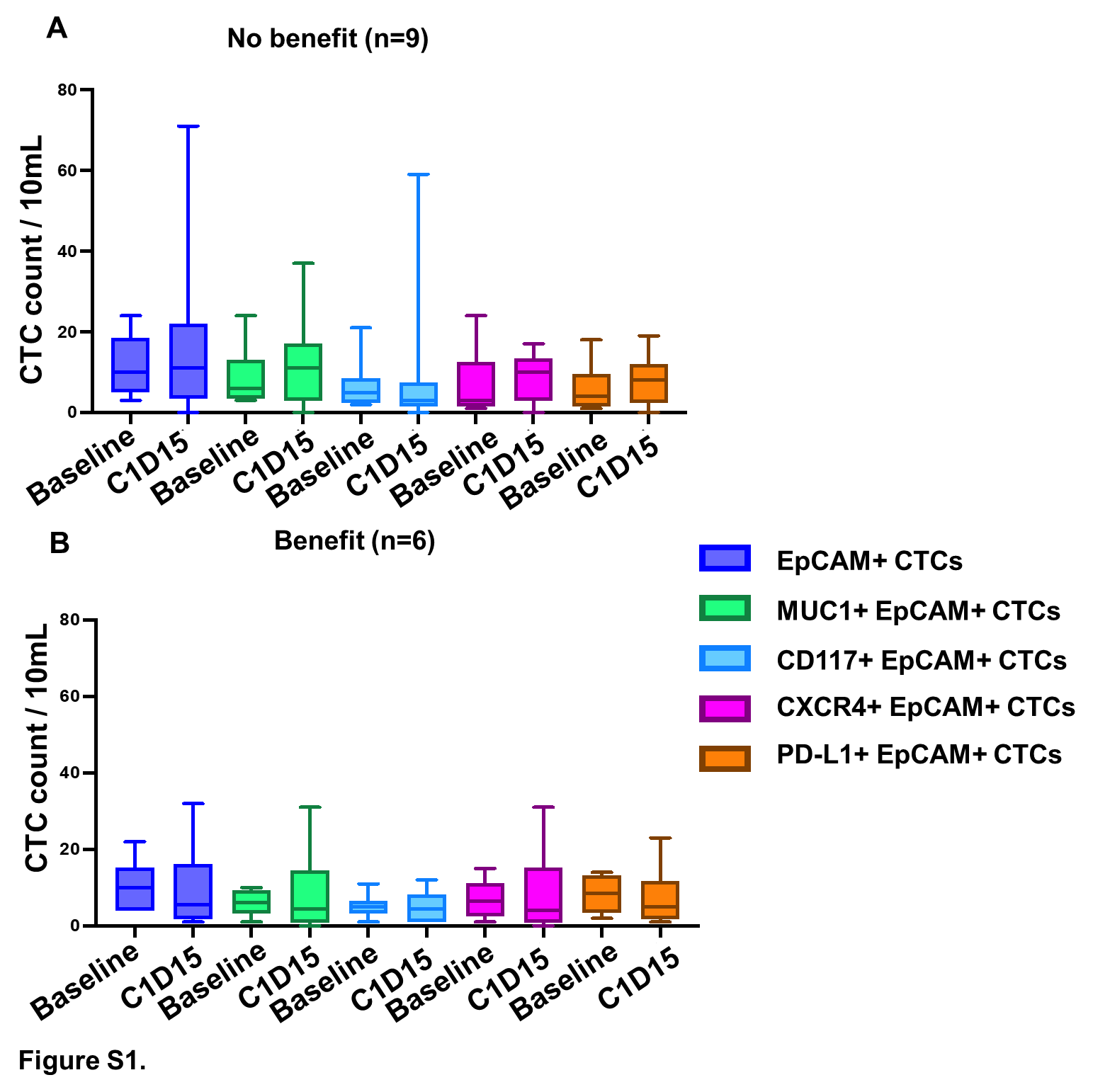


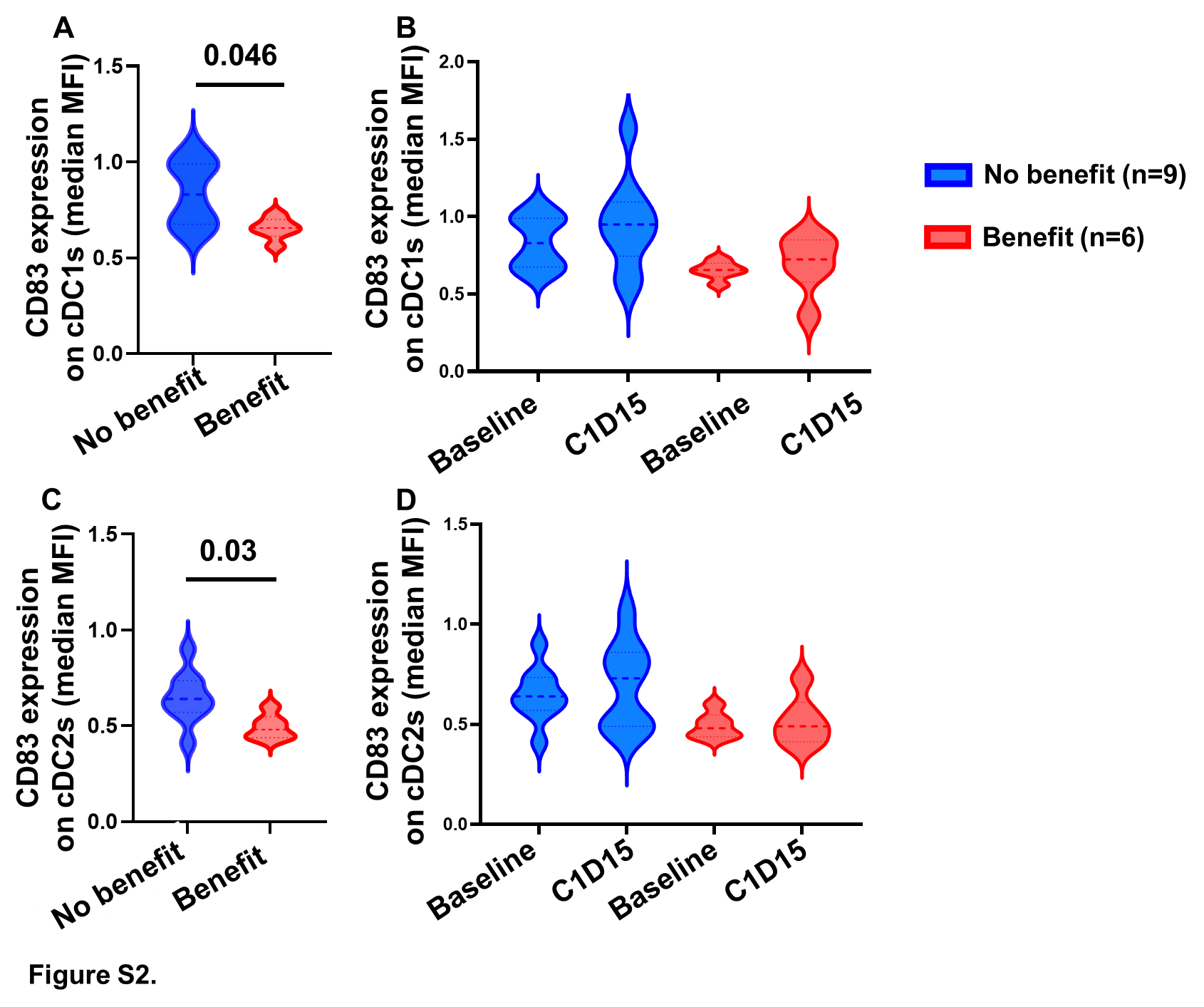


**
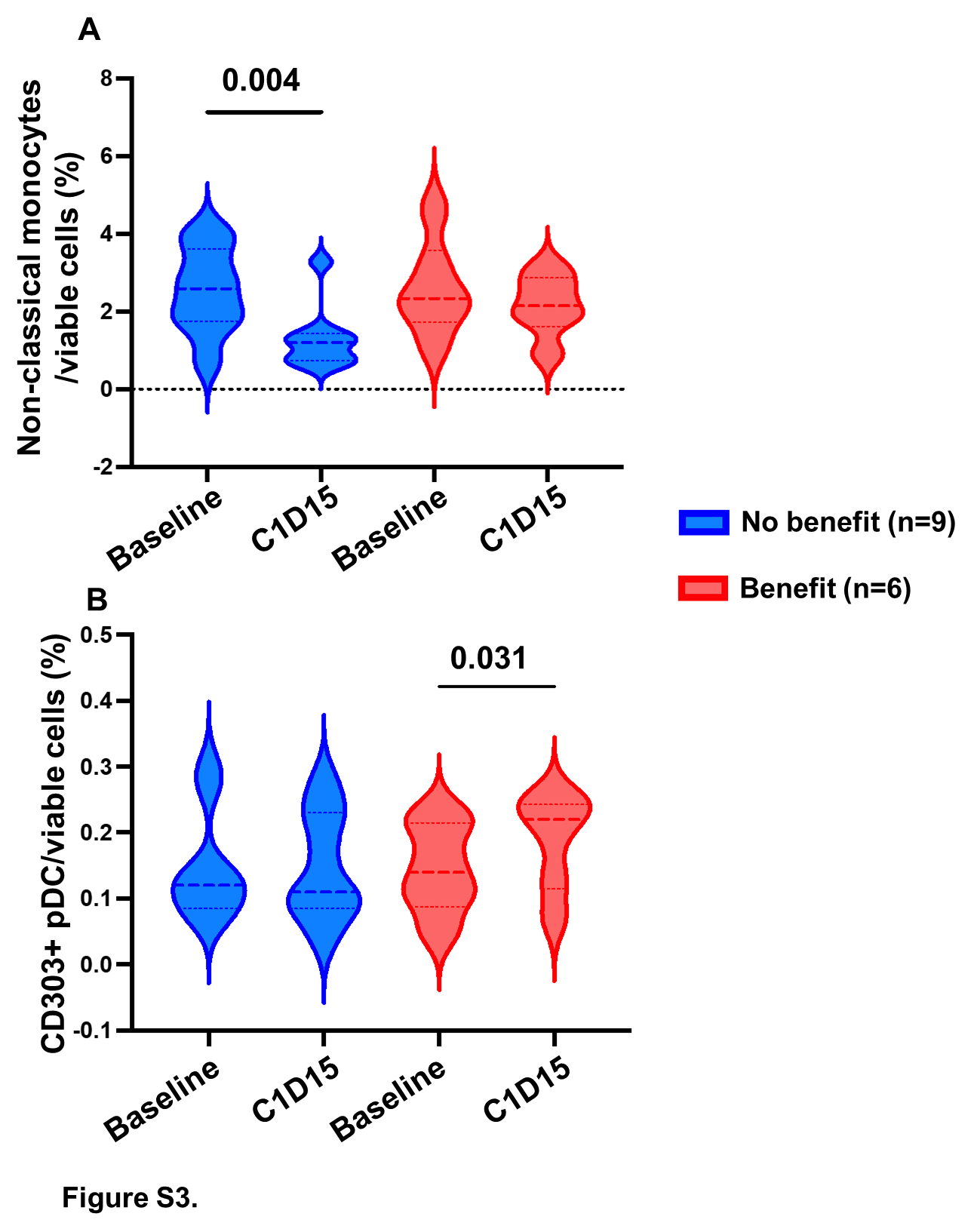
**

**
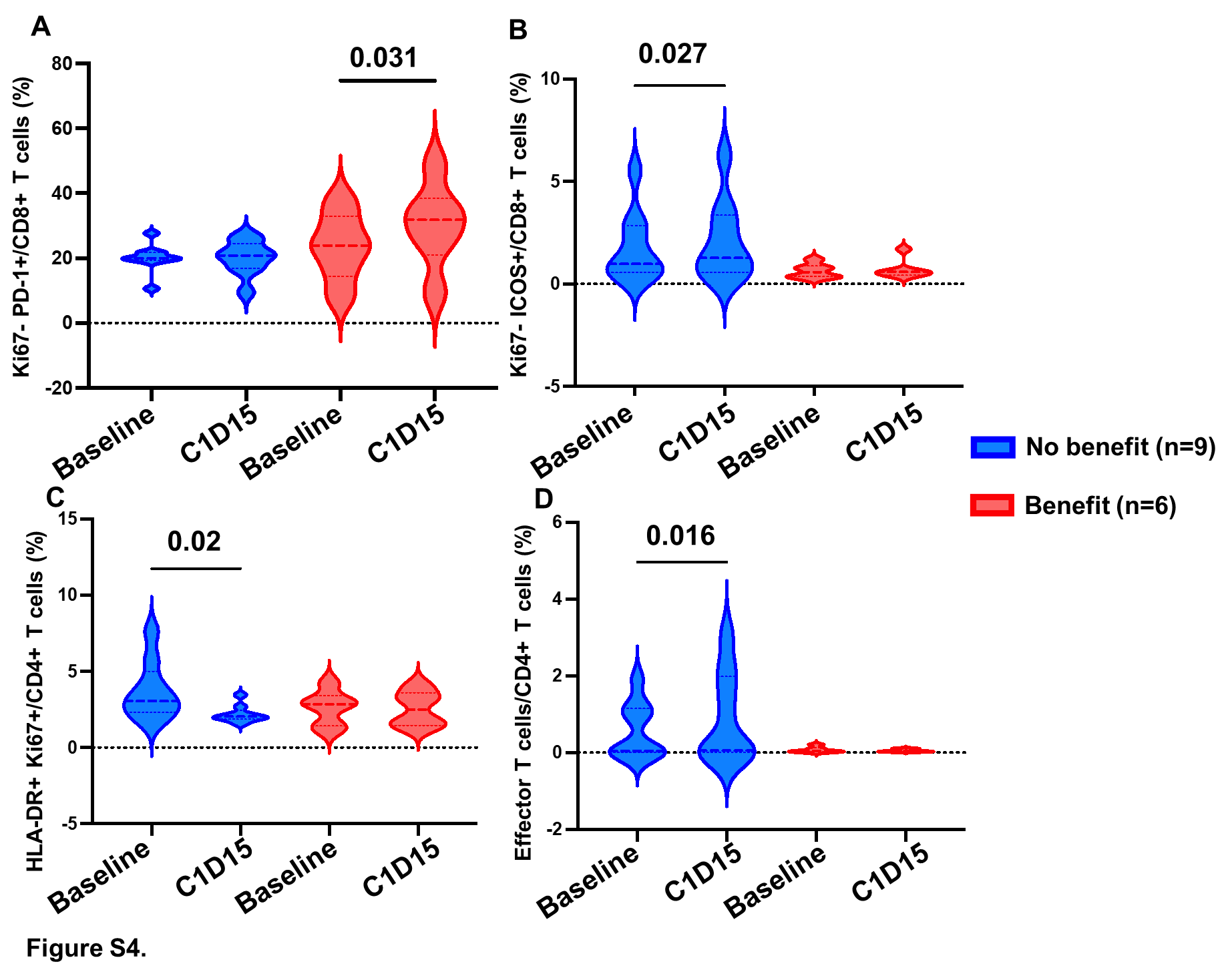
**

**Data S1. Inclusion and Exclusion criteria**

*Inclusion criteria*

1. Patients must have histologically confirmed persistent or recurrent triple-negative breast cancer (TNBC).
2. Documentation of germline *BRCA1* and *BRCA2* mutation (gBRCAm) status will be requested for eligibility. Patients with VUS or deleterious mutation in other genes without gBRCAm or patients with negative BRCA testing are still eligible.
3. Patients must have measurable diseases defined by RECIST v1.1 as those that can be accurately measured in at least one dimension (longest diameter to be recorded) as:

• By chest x-ray: >20 mm;

• By CT scan:

o Scan slice thickness 5 mm or under as >10 mm with CT scan

o Scan slice thickness >5 mm: double the slice thickness

• With calipers on clinical exam>10 mm.

1. Patients must have at least one lesion deemed safe to biopsy and be willing to undergo a mandatory baseline biopsy.
2. Age >18 years.
3. Eastern Cooperative Oncology Group (ECOG) performance status ≤ 2.
4. Patients must have adequate organ and marrow function as defined below:

- absolute neutrophil count ≥ 1,500/mcL
- white blood cell (WBC) > 3,000/mcL
- platelets ≥ 100,000/mcL
- hemoglobin (Hgb) ≥ 9 g/dL in the absence of packed red blood cell transfusion 28 days prior to dosing
- AST(SGOT)/ALT(SGPT) ≤ 2.5 X institutional upper limit of normal (ULN); for subjects with liver metastases, AST or ALT ≤ 5 × ULN a. Total bilirubin ≤ 1.5 × ULN; for subjects with documented/suspected Gilbert’s disease, bilirubin ≤ 3 × ULN
- creatinine ≤ 1.5 X within normal institutional limits

OR

measured creatinine clearance > 50 mL/min/1.73 m^2^

1. Toxicities of prior therapy (excepting alopecia) should be resolved to less than or equal to Grade 1 as per CTCAE v 4.03 (http://ctep.cancer.gov/protocolDevelopment/electronic_applications/ctc.htm) except hemoglobin as shown above. Patients with long-standing stable grade 2 neuropathy may be considered after discussion with the principal investigator (PI).
2. Female patients must either be of non-reproductive potential (i.e., post-menopausal by history: ≥60 years old and no menses for ≥1 year without an alternative medical cause; OR history of hysterectomy, OR history of bilateral tubal ligation, OR history of bilateral oophorectomy) or must have a negative serum pregnancy test upon study entry and agree to use contraception or abstinence for female patients with reproductive potential.

*Exclusion criteria*

1. Patients who have received chemotherapy in the previous 3 weeks (6 weeks for nitrosoureas or mitomycin); or who received radiotherapy or any other investigational agents within 3 weeks prior to study enrollment.
2. Patients who have received prior PARP inhibitors (PARPi) are ineligible. For this study, BSI-201 (iniparib) is not considered as PARPi.
3. Patients who have had durvalumab. Prior treatment with other immune checkpoint inhibitors is allowed.
4. Patients receiving any medications or substances that are strong inhibitors or inducers of CYP3A4.
5. Major surgical procedure within 30 days prior to the first dose of durvalumab or still recovering from prior surgery.
6. Patients with any other concomitant or prior invasive malignancies are ineligible except for prior cancer treated with a curative intent with no evidence of recurrent disease 5 years following diagnosis and judged by the investigator to be at low risk of recurrence and treated limited stage basal cell or squamous cell carcinoma of the skin or carcinoma in situ of the breast or cervix.
7. Patients with evidence of CNS metastasis, spinal cord compression, or leptomeningeal disease within six months prior to enrollment. Patients with a remote history of brain metastases may be considered if they received sterilizing therapy to the CNS (resection or radiation) and have been CNS progression-free for the six months period. Patients with spinal cord compression may be considered if the condition is medically managed and currently asymptomatic.
8. History of allergic reactions attributed to compounds of similar chemical or biologic composition to durvalumab, olaparib or to other humanized monoclonal antibodies. Known history of anaphylaxis, angioedema, laryngeal edema, serum sickness, or uncontrolled asthma.
9. History of auto-immune disease requiring steroid maintenance, or history of primary immunodeficiency.
10. Current or prior use of immunosuppressive medication within 28 days before the first dose of durvalumab, except for intranasal and inhaled corticosteroids or systemic corticosteroids at physiological doses, which are not to exceed 10 mg/day of prednisone or an equivalent corticosteroid. In the case of short-term use of systemic corticosteroids (less than 24 hours within 28 days) of greater than 10 mg/day of prednisone or an equivalent corticosteroid, the required washout period prior to starting the first dose of durvalumab is 7 days.
11. Active or prior documented inflammatory bowel disease (e.g., Crohn’s disease, ulcerative colitis). Eligibility for patients with asymptomatic and a previous diagnosis of immune or inflammatory colitis, or patients with chronic diarrhea > 1 month without immune or inflammatory colitis is a PI decision on an individual patient basis.
12. Receipt of live attenuated vaccination within 30 days before the first dose of durvalumab.
13. Patients with any cardiac history of the following conditions within 1 year prior to study enrollment are excluded from the study:
    - Prior events including myocardial infarction, clinically significant pericardial effusion, and myocarditis.
    - Prior cardiac arrhythmia including atrial fibrillation (except chronic atrial fibrillation with controlled vascular rate) and atrial flutter or requiring concurrent use of drugs or biologics with pro-arrhythmic potential.
    - NYHA Class II or greater heart failure.
    - If cardiac function assessment is clinically indicated or performed, an LVEF less than normal per institutional guidelines, or <55%, if threshold for normal is not otherwise specified by institutional guidelines.
    - Mean QT interval corrected for heart rate (QTc) ≥470 ms calculated from 3 electrocardiograms (ECGs) using Frediricia’s Correction or other significant ECG abnormality noted within 14 days of treatment.
    - • Clinically significant peripheral vascular disease or vascular disease, including rapidly growing aortic aneurysm or abdominal aortic aneurysm >5 cm or aortic dissection.
    - • Unstable angina.
14. History of cerebrovascular accident, transient ischemic attack within 1 year prior to study enrollment.
15. Significant hemorrhage (> 30 mL bleeding/episode within 3 months before study enrollment) or hemoptysis (> 5mL fresh blood within 28 days before study enrollment).
16. Current dependency on Total parenteral nutrition or intravenous fluid hydration.
17. Any prior grade ≥ 3 immune-related adverse event (irAE)while receiving any previous immunotherapy agent, or any unresolved irAE > grade 1. Toxicities of prior therapy, excepting alopecia, should be resolved to less than or equal to grade 1 as per NCI-CTCAEv4.0 (located on the CTEP website at http://ctep.cancer.gov/protocolDevelopment/electronic_applications/ctc.htm).
18. Uncontrolled intercurrent illness including, but not limited to, ongoing or active infection, symptomatic congestive heart failure, unstable angina pectoris, cardiac arrhythmia (except chronic atrial fibrillation with controlled vascular rate), active peptic ulcer disease, or psychiatric illness/social situations that would limit compliance with study requirements.
19. Pregnant and breastfeeding women are excluded from this study.
20. HIV-positive patients on antiretroviral therapy are ineligible because of potential pharmacokinetic interactions with study drugs. However, patients with long-standing (>5 years) HIV on antiretroviral therapy > 1 month (undetectable HIV viral load and CD4 count > 150 cells/μL) may be eligible if the PI determines no anticipated clinically significant drug-drug interactions.
21. HBV-or HCV-positive patients are ineligible because of potential reactivation of hepatitis virus following steroids.
22. Known history of previous clinical diagnosis of tuberculosis.
23. No baseline features suggestive of myelodysplastic syndrome or acute myelogenous leukemia on peripheral blood smear or bone marrow biopsy, if clinically indicated.
24. No prior or current evidence of coagulopathy or bleeding diathesis. Therapeutic anticoagulation for prior thromboembolic events is permitted.
25. Concurrent enrollment in another clinical study, unless it is an observational non-interventional clinical study or the follow-up of an interventional study.
26. Any concurrent chemotherapy, immunotherapy, biologic or hormonal therapy for cancer treatment. Concurrent use of hormones for non-cancer-related conditions (e.g., insulin for diabetes and hormone replacement therapy) is acceptable. NOTE: Local treatment of isolated lesions for palliative intent is acceptable (e.g., by local surgery or radiotherapy).

**Data S2. Dosing delays/dose modifications and management of toxicities**

Dose delays and modifications will be made using the following recommendations.

1. *General Recommendation for Dose Modification*

| **Dose Level** | **Olaparib tablets** |
| --- | --- |
| 1 | 300 mg twice daily |
| -1 | 250 mg twice daily |
| -2 | 200 mg twice daily |

| **Dose Level** | **Durvalumab IV** |
| --- | --- |
| 1 | A fixed dose of 1500 mg every 4 weeks |
| -1 | A fixed dose of 500 mg every 4 weeks |

1. *General Recommendations for Management of Hematologic Adverse Events*

Treatment may be delayed for a maximum of 14 days after holding the treatment for toxicities that develop and do not resolve as defined in the protocol. For absolute neutrophil count (ANC) ≥ 1000/mcL, platelets ≥ 75,000/mcL, hemoglobin ≥ 8 mg/dL, no interruption was initiated. For ANC < 1000/mcL or platelets < 75,000/mcL or hemoglobin < 8 mg/dL, on the first occurrence, the drug(s) were held for up to 14 days, on the second occurrence, appropriate medical therapy was initiated. For grade 4 hematologic AEs, on the first occurrence, appropriate medical therapy was initiated, and the drug(s) were held for up to 14 days, and on the second occurrence, the drug(s) causing the toxicity was discontinued.

- Growth factors to prevent neutropenia will not be administered prophylactically but can be used during a drug hold to assist the recovery. Filgrastim/PEG filgrastim will be used only if the patient has neutropenia with sepsis for the purpose of facilitating recovery of the neutropenic sepsis.
- Symptomatic anemia should be treated with red blood cell transfusion and is recommended if the hemoglobin falls below 8 g/dL or the patient is symptomatic. The initiation of erythropoietic therapy for the management of chemotherapy-induced anemia follows the American Society of Hematology/ASCO clinical practice guidelines (<http://www.asco.org>).

1. *General Recommendations for Management of Non-Hematologic Adverse Events*

- For grade 1 and 2 AEs that resolves within 48 hours with supportive care, dose modification was not required. For any ≥ grade 3 non-hematologic, hold study drug(s) for up to 14 days until toxicity resolves to ≤ grade 1. Treatment with olaparib may be restarted at one DL lower. For grade 3 or 4 AEs related to durvalumab and olaparib combination therapy that do not resolve to grade 1 or less within 14 days despite maximum intervention after treating patient at the lowest reduced DL, the patient was removed from the study.
  1. Durvalumab

3.1.1 Infusion-related reaction with durvalumab

In the event of grade 1 or 2 infusion-related reaction, the infusion rate of study drug may be decreased by 50% or interrupted until resolution of the event (up to 4 hours) and re-initiated at 50% of the initial rate until completion of the infusion. For patients with grade 1 or 2 infusion-related reaction, subsequent infusions may be administered at 50% of the initial rate. Acetaminophen and/or an antihistamine (e.g., diphenhydramine) or equivalent medications may be administered at the discretion of the PI. If the infusion-related reaction is grade 3 or higher OR recurrent grade 2 or higher in severity, Durvalumab will be discontinued and olaparib will continue the maintenance do

3.1.2 Immune-mediated adverse events

- For grade 1, study regimen was continued unless clinically appropriate and no dose modification was made. For grade 2, study regimen dose was held until the resolution to ≤ grade 1. For grade 3 and 4 pneumonitis/interstitial lung disease, diarrhea and enterocolitis, empiric IV methylprednisolone 1 to 4 mg/kg/day or equivalent was promptly initiated, and the study regimen dose was permanently discontinuation. For grade 3 hepatitis (Elevated LFTs), rash (excluding bullous skin formations), endocrinopathy (e.g., hyperthyroidism, hypothyroidism, hypopituitarism, adrenal insufficiency, etc.), immune mediated neurotoxicity, study regimen dose was held until the resolution to ≤ grade 1. For grade 4, hepatitis (Elevated LFTs), rash (excluding bullous skin formations), endocrinopathy (e.g., hyperthyroidism, hypothyroidism, hypopituitarism, adrenal insufficiency, etc.), immune mediated neurotoxicity, the study regimen dose was permanently discontinuation.
